# Quality improvement intervention to increase adherence to ART prescription policy at HIV treatment clinics in Lusaka, Zambia: A cluster randomized trial

**DOI:** 10.1371/journal.pone.0175534

**Published:** 2017-04-18

**Authors:** Elizabeth A. McCarthy, Hamsa L. Subramaniam, Margaret L. Prust, Marta R. Prescott, Felton Mpasela, Albert Mwango, Leah Namonje, Crispin Moyo, Benjamin Chibuye, Jan Willem van den Broek, Lindsey Hehman, Sarah Moberley

**Affiliations:** 1 Applied Analytics, Clinton Health Access Initiative, Lusaka, Zambia; 2 Applied Analytics, Clinton Health Access Initiative, Boston, Massachusetts, United States of America; 3 Demand-Driven Evaluations for Decisions, Clinton Health Access Initiative, Lusaka, Zambia; 4 Clinical Care and Diagnostic Services, Ministry of Health, Lusaka, Zambia; 5 Mother and Child Health, Ministry of Community Development, Mother and Child Health, Lusaka, Zambia; 6 Clinton Health Access Initiative, Lusaka, Zambia; 7 Health Financing, Clinton Health Access Initiative, Lusaka, Zambia; 8 Applied Analytics, Clinton Health Access Initiative, Kampala, Uganda; Public Library of Science, FRANCE

## Abstract

**Introduction:**

In urban areas, crowded HIV treatment facilities with long patient wait times can deter patients from attending their clinical appointments and picking up their medications, ultimately disrupting patient care and compromising patient retention and adherence.

**Methods:**

Formative research at eight facilities in Lusaka revealed that only 46% of stable HIV treatment patients were receiving a three-month refill supply of antiretroviral drugs, despite it being national policy for stable adult patients. We designed a quality improvement intervention to improve the operationalization of this policy. We conducted a cluster-randomized controlled trial in sixteen facilities in Lusaka with the primary objective of examining the intervention’s impact on the proportion of stable patients receiving three-month refills. The secondary objective was examining whether the quality improvement intervention reduced facility congestion measured through two proxy indicators: daily volume of clinic visits and average clinic wait times for services.

**Results:**

The mean change in the proportion of three-month refills among control facilities from baseline to endline was 10% (from 38% to 48%), compared to a 25% mean change (an increase from 44% to 69%) among intervention facilities. This represents a significant 15% mean difference (95% CI: 2%-29%; *P* = 0.03) in the change in proportion of patients receiving three-month refills. On average, control facilities had 15 more visits per day in the endline than in the baseline, while intervention facilities had 20 fewer visits per day in endline than in baseline, a mean difference of 35 fewer visits per day (*P* = 0.1). The change in the mean facility total wait time for intervention facilities dropped 19 minutes between baseline and endline when compared to control facilities (95% CI: -10.2–48.5; *P* = 0.2).

**Conclusion:**

A more patient-centred service delivery schedule of three-month prescription refills for stable patients is viable. We encourage the expansion of this sustainable intervention in Zambia’s urban clinics.

## Introduction

The global HIV response continues to evolve as the quality of diagnostics and antiretroviral drugs improve and become increasingly accessible to a growing number of patients worldwide [1–3]. Addressing the HIV epidemic in cities is a global priority, with the rapid urbanization in Africa and Asia, home to 83% of all people living with HIV [4]. Many countries in sub-Saharan Africa, including Zambia, are shifting from an emergency response to HIV towards a longer-term sustainable response with increased attention on efficiency, effectiveness, and quality of care. HIV programs seek to identify patients as soon as possible after infection, offer them the best antiretroviral drugs available, and provide the routine clinical, psychosocial and diagnostic services they need for their long-term health. Expanded HIV treatment eligibility criteria, and these improvements in access to drugs and diagnostics has led to a growing number of patients on antiretroviral treatment in Zambia and elsewhere [5].

Treatment programs do face challenges, however, when patient volumes at the health facility outpace the growth of the health workforce available to care for them and the health facility infrastructure to accommodate them. Particularly in urban areas such as Zambia’s capital city, Lusaka, the result can be crowded facilities with long patient wait times [6], which can be deterrents for patients to attend their clinical appointments and pick up their medications [7–13]. Whereas in rural areas, patients struggle more with having to walk long distances to get to a health facility, patients in cities such as Lusaka typically travel a shorter distance to reach the health facility, but struggle once they arrive with crowds and long wait times. Facility congestion can, ultimately, disrupt patient care and compromise patient retention and adherence.

As the methods to improve facility congestion are multi-faceted, formative research was necessary to develop a contextually appropriate intervention. Therefore, we first performed formative research to get at the root causes of HIV treatment clinic congestion. We used these findings to design an intervention to address the root causes, and then performed a cluster-randomized controlled trial to determine the impact of the intervention on HIV treatment clinic congestion. Our primary objective was to examine the impact of a quality improvement intervention on the proportion of stable patients receiving three-month refills. The secondary objective was examining whether the intervention reduced facility congestion measured through two proxy indicators: daily volume of clinic visits and average clinic wait times for services. We hypothesized that the intervention would increase fidelity to the three-month refill policy, leading to reduced patient wait times and smaller patient load, and ultimately improved patient retention and health outcomes. After ascertaining the impact of the intervention, we were able to recommend whether it should be expanded to the rest of Lusaka and other urban areas of Zambia.

## Materials and methods

### Formative research

To inform the design of the intervention, we conducted formative research at eight randomly selected ART clinics in Lusaka, Zambia, in November 2014 [14]. To document the client flow and amount of time spent waiting for services, we directly observed patient wait time and time spent receiving care at each clinic station for 80 adult ART patients (ten per facility). To understand the pattern of refills for each facility, we reviewed ART registry records for 80 stable patients (ten per facility) and examined the proportion of stable patients receiving one-month, two-month, and three-month refills. A patient receiving a one-month supply of drugs would be required to visit the facility twelve times a year to pick up their drugs. A patient receiving a two-month supply would need six pharmacy visits, while a patient receiving a three-month refill supply would need to visit the pharmacy four times a year. To be characterized as stable, a patient must have been on first-line treatment for more than six months, have no health conditions requiring clinical attention, and have not switched medication in the last three months. To assess whether the proportion of three-month refills was associated with stockouts, we obtained stockout history of antiretroviral drugs (ARVs) from medical store registers and examined the stockout pattern vis-à-vis the proportion of stable patients receiving three-month refills. As an exploratory assessment, all analyses were descriptive and no statistical tests were performed.

As of 2013, Zambia’s national policy has been to give a three-month supply of antiretroviral drugs to stable HIV treatment patients, in line with the World Health Organization’s policy of multi-month prescriptions for stable patients [2, 15]. Nonetheless, we found that three-month refills were not being given to many eligible patients. An average of only 46% of stable patients across the eight facilities received a three-month supply of ARVs, with wide variation across sites (between 8% and 70%).

We found that almost half (47.5%) of all patients at the facility were there only to refill their prescription at the pharmacy. These “refill” patients spent an average of one hour and 34 minutes from the first encounter in the registration area to departure, not including additional time spent waiting to be called to the registration desk (registered) before their appointment.

Given the challenges of clinic congestion to Lusaka’s patient population, and the existence of a national policy that, if adhered to, could decrease the number of times in a year that a patient needed to return to the clinic, we designed a quality improvement (QI) intervention in consultation with the Lusaka District health team to improve the operationalization of the three-month drug refill policy. Evidence in support of QI interventions to improve HIV treatment services comes from places such as Canada and Ethiopia where systems-level approaches have been taken to improve health service delivery [16, 17].

### Intervention description

The intervention package included several components that empowered district health officers and facility in-charges with support, guidance and tools to ensure stock availability and appropriate antiretroviral drug dispensing practices. All study sites (control and intervention alike) received a policy memo directing staff to follow the MOH policy to provide three-month refills. All study sites received support from the Lusaka District Medical Office (DMO) in stock planning to meet the expected initial surge in the demand for drug stocks (S3 File).

A key element that existed at the intervention facilities and not the control facilities was a quality improvement officer. An existing member of staff at each intervention facility was assigned as a QI officer for that facility; their mandate was to support the provision of three-month refills. They were equipped with guidance materials and checklists to administer at each facility at the beginning of the intervention (S4 File) and on a weekly basis thereafter (S5 File). The QI officers used the materials to monitor drug and lab stock levels, ensure health care worker implementation of the three-month refill policy, record and troubleshoot challenges faced by the facility, and communicate those to the district level for resolution. Intervention facilities also received a job aide in the form of a poster targeted towards reminding pharmacists of the three-month refill policy and taking away the decision-making step at the time of drug dispensation (S1 File); the full amount was to be given or the reason why it was not possible was to be discussed with the QI officer.

A district-level intervention manager was appointed from within the existing staff at the Lusaka DMO to resolve those challenges beyond the QI officers’ capability. The intervention manager visited intervention sites weekly and reviewed checklists completed by the QI officers weekly. The intervention manager and QI officers were provided with training, funds for transport and troubleshooting, and a small monthly stipend. Trainings occurred on January 29, 2015, and the intervention began at facilities on February 1, 2015.

The intervention ran for three months at an average of $475 per month per facility (based on the Zambia Kwacha translated to US Dollar prices at the 180-day average exchange rate at July 1, 2015 of ZMW 7.0607) (S2 File). The majority (69%) of the costs were operational and included communication, transport, printing, fuel, and flexible funds for facility implementation (used at the discretion of the facility to address observed delivery challenges). The next largest cost category (30%) was QI officer and intervention manager incentives, followed by a small expenditure (1%) for training costs.

### Trial design

We designed a cluster-randomized controlled trial (cRCT). As mentioned above, the primary objective was to examine the impact of the quality improvement intervention on the proportion of stable patients receiving three-month refills. The secondary objective was examining whether the QI intervention reduced facility congestion measured through two proxy indicators: daily volume of clinic visits and average clinic wait times for services.

#### Sampling and randomization

Primary sampling units for the cRCT were public sector ART facilities within Lusaka District not currently engaged in another study or intervention. Facilities were excluded if they were private or not using SmartCare, the MOH electronic medical records system that maintains detailed patient records of all ART and pre-ART patients of participating health facilities in Lusaka District, including patient history, historical appointment records, clinical results, lab results, and prescription history [18].

The study population within selected facilities was stable, HIV-infected adult treatment patients. Stable patients were defined as those that were on first-line HIV treatment drug regimens as recommended by the MOH [15], had been on treatment and consistently retained for more than six months as of October 1, 2014, and had no other health conditions (e.g., opportunistic infections) requiring attention of a clinician. Such patients were eligible for a three-month refill as outlined in Zambia’s 2014 HIV Treatment Guidelines [15].

Sixteen facilities were necessary to properly power the study. With an average of 2,300 patients per facility and a 20% potential for missing records, we had an 84% power (β = 0.84) and 95% confidence interval (CI) (α = 0.05) to detect a difference of 17% or more in the change in the proportion of patients receiving three-month refills from baseline to endline in the intervention group against the change in the control group. For the sample size calculation, we used June 2014 data to estimate the parameters, a change of five percent in the control group, and assumed that all eligible patient records would be acquired [19]. Without a pre-defined rho estimate, we considered 0.1 as a reasonable value based on current scholarship [20]. All estimates were checked once baseline data was gathered.

Eligible facilities were each assigned a computer-generated random number by the study team using Excel, out of which the 16 facilities with the lowest random number were selected. The proportion of patients receiving three-month refills was then calculated for each facility and sorted in descending order. Facilities were then pair-matched with the closest proportion value. Facilities within each pair were again assigned a computer-generated random number and selected the lowest number within a pair to the intervention. Facilities were not blinded to their status as an intervention or control site.

### Data collection

The baseline period was October 1—December 31, 2014, and the endline period was February 1—April 30, 2015. De-identified ART registry data from all participating facilities was collected from SmartCare on a rolling basis to include patient data from October 2014—April 2015.

Patient visit times were collected once during baseline and once during endline via time-motion observations at each facility. Trained data collectors visited each facility and recorded on a paper form the arrival and departure time of randomly identified adult patients as they moved among clinic stations during their clinic visit. This allowed the calculation of both wait time and service time for each tracked patient. Patients were selected through systematic random sampling until the facility sample size was achieved. Patients were not aware of the data collection.

Stock data were collected at each study facility from October 2014—April 2015. Data collectors visited each study facility between May 1—May 15, 2015 and used camera phones to photograph the stock balance card for each first-line drug to provide a continuous count of the number of tablets in stock at the facility for the duration of the study period. The data from the stock card photographs were entered into a database, providing insight into stock replenishment frequency, stock-out frequency and duration, and first-line treatment volumes.

For patient visit time, facility characteristics and stock data, baseline data were collected from February 1 to 15, 2015. Endline data were collected from April 20—May 5, 2015. The data were collected as cross-sectional segments at baseline and endline for all facilities; participants were not followed over time.

### Outcomes

The primary outcome was the change in the proportion of stable ART patients receiving three-month refills from baseline to endline, calculated as the absolute difference in the change of the proportion of patients receiving three-month refills among stable patients between intervention and control facilities. Under the SmartCare electronic record system, this primary outcome was defined as the stable patients who attended at least one appointment and ever received a three-month refill during a three-month period (baseline and endline). We categorized a three-month prescription as those patients who received the appropriate number of tablets needed for 90 days as per their prescribed regimen. For example, a fixed-dose combination requires one tablet per day, so the patient would have received 90 pills for three months. If the prescription required two tablets per day, a three-month prescription would be 180 tablets.

Secondary outcomes included: 1) the change in patient wait time, measured as the absolute difference in the average patient wait time from the triage nursing station to exiting the facility between intervention and control facilities; and 2) the change in daily patient load, measured as the absolute difference in the change in average daily number of patients attending intervention and control facilities. Wait time was defined as the number of minutes between leaving one station and when a patient was called at the next station. Daily patient load was calculated using appointment history as recorded in SmartCare during the baseline and endline periods.

Facility first line ARV stock levels, assessed by the number and duration of stock-outs or low stock incidents in each of the baseline and intervention periods, were measured as a specific factor enabling intervention success. Facility stock levels were also measured as an exploratory outcome.

### Statistical analysis

Baseline differences in facility-level characteristics between the two arms were described using t-tests. Any factors that differed at baseline were considered for the adjusted analyses; regardless, final factors included in the analysis were those that were theoretically thought to be confounders between the intervention and outcome.

As estimates of the proportion of stable patients receiving three-month refills were used to match; matching efficiency was assessed using baseline data from the three month-period of October to December 2014 to determine whether the pair-matched analysis was appropriate. Matching was deemed efficient if the pairwise correlation on three-month refills was significantly greater than 0.2 [21, 22]. After initial assessment, the matching was not significantly greater than 0.2, and therefore, matching was not kept during the analysis.

For the primary outcome, we calculated mean change in the proportion of patients receiving three-month refills between baseline and endline for each facility (i.e. cluster summary of change). For the secondary outcomes, the absolute difference in the change in wait times between the intervention and control facilities (difference-in-differences) was conducted. Additionally, after considering baseline data, analysis examining the change in the ratio of the wait time to processing time for the intervention group was included.

For both the primary and secondary outcomes, we ran analyses using facility-level summary measures, recognized as the most robust analysis method given the small number of clusters per arm (<10) and the fact that the data was cross-sectional at the facility level [23]. For the primary outcome, we calculated the mean change between baseline and endline for each facility (i.e., cluster summary of change). We then calculated weighted and unweighted pooled summary measures of change for each arm. To compare these summary measures of change between the two arms we used unweighted t-tests (difference-in-difference). We tested the robustness of the t-test by performing ln-transformed unweighted t-tests and non-parametric comparisons. Additionally, to adjust for covariates, we conducted t-tests on the residuals of our outcomes resulting from generalized linear regression models. The linear regressions predicted the change in our outcome dependent on our theoretical confounders and excluded our intervention and facility-level clustering. The residuals from this model were then summarized per facility and compared using t-tests [23]. We considered weighting the cluster summaries for the t-tests, however there is no clear consensus on how best to apply weights to account for the variance of the clusters when analyzing the change at the facility-level from cross-sectional repeated measures [19, 24]. Additionally, if there are few clusters and rho is not precisely calculated, the use of weights may actually result in a loss of efficiency and potentially incorrect conclusions. Given the lack of accuracy when constructing weights, unweighted tests were selected for the final analysis.

For the secondary outcome, we similarly completed a comparison of the absolute difference in the change in wait times between the intervention and control facilities. Additionally, based on discussions of the baseline data, we included an analysis examining the change in the ratio of the wait time to processing time for the intervention group.

As a final sensitivity test for all outcomes, we also ran multi-level analyses examining the relation between the intervention and individual outcomes at endline accounting for facility-level characteristics (i.e., baseline values) using generalized estimation equations (GEE).

To examine whether stock levels impacted the ability of health facilities to provide three-month refills, stock levels were descriptively analysed continuously over the duration of the study period for each first-line drug. We also tabulated incidents of stock-outs, defined as when the stock level for a particular drug was zero for two or more days.

All quantitative analyses were conducted in Stata, version 13.

### Ethical considerations and trial registration

This study received approval on September 19, 2014, from Excellence in Research Ethics and Science (ERES) Converge IRB, a private institutional review board in Lusaka, Zambia (S1 Protocol). The amended protocol received approval on January 19, 2015 (S2 Protocol). For all forms of data collection where direct interaction with participants was involved, including the interviews and focus groups, written informed consent was obtained prior to data collection. As approved by the IRB, informed consent was not obtained for participation in the study related to the primary analysis of proportion of patients receiving 3-month refills, or secondary analysis of patient wait times since patients received care from health workers from the facility performing routine care functions. Further, primary analyses were conducted using de-identified patient data aggregated to the facility level, the unit of randomization, and therefore no informed consent from individual patients was required. This study was retrospectively (due to oversight of the requirement at the time of enrolment) registered with the Pan African Clinical Trial Registry (PACTR) on August 4, 2015 under identification number PACTR2015008001148419. The authors confirm that there are not any ongoing or related trials requiring registration.

## Results

**Fig 1:**
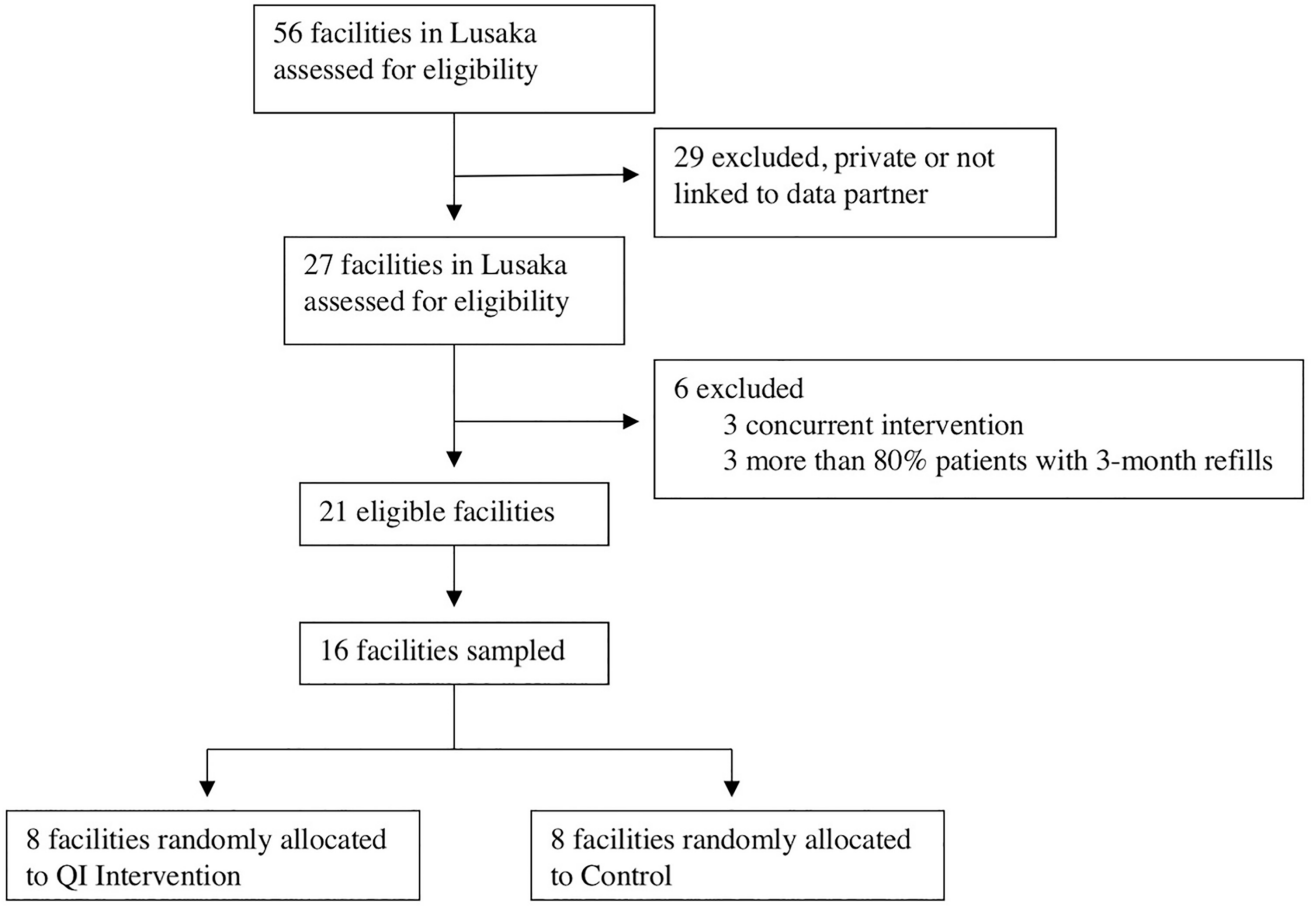
Trial profile.

Twenty-seven public facilities in Lusaka were assessed for eligibility. Three facilities were excluded because the proportion of patients receiving three-month refills estimated from June 2014 data exceeded 80%. An additional three facilities were excluded because they were enrolled in another HIV service-delivery study. Of the remaining 21 facilities, 16 were randomly selected for the study and pair-matched based on their estimated proportion of patients on three-month refills (Fig 1).

In baseline, data from 9,470 stable patients in control sites and 8,258 in intervention sites were analysed. Patient and facility characteristics were not significantly different between groups in either the baseline or endline, as evidenced by the t-test p-values in Table 1. Twenty patients from each facility during the baseline and endline data collection were included in the analysis of patient visit time.

**Table 1:** Baseline and endline characteristics of facilities by treatment group.

	Baseline			Endline		
	Control	Intervention	*t-test p-value*	Control	Intervention	*t-test p-value*
Adult ART Facility Patient Population						
Total Number of Active ART Patients	32,762	30,541		41,824	32,358	
Total Number of Stable ART Patients	9,470	8,258		9,633	7,879	
Adult ART Patients, ALL *						
Per facility, mean	4,095	3,818	*0*.*80*	5,228	4,044	*0*.*38*
Visits per day per facility, mean	311	286	*0*.*72*	334	269	*0*.*37*
Adult ART Patients, Stable *						
Per facility, mean	1,184	1,032	*0*.*63*	1,204	984	*0*.*49*
Visits per day per facility, mean	41	35	*0*.*57*	41	29	*0*.*22*
Human Resources, Mean Staff Number by cadre per facility*†						
Registered Nurse (3 years of training)				2	3	*0*.*44*
Enrolled Nurses (2 years of training)				5	3	*0*.*11*
Clinical Officers				2	3	*0*.*70*
Doctors				1	1	*0*.*40*
Pharmacists				2	3	*0*.*48*
Lab Staff				2	3	*0*.*73*

*All means rounded to the nearest integer in reference to patients

^†^ Human resources data was assumed to not change significantly over the eight-month observation period and therefore were collected at the end of the study; this assumption was programmatically confirmed.

### Impact of intervention on three-month refills

The mean change in the proportion of three-month refills among control facilities from baseline to endline was 10% (from 38% to 48%), compared to a 25% mean change (an increase from 44% to 69%) among intervention facilities. This represents a significant 15% mean difference (95% CI: 2%-29%; *P* = 0.03) in the change in proportion of patients receiving three-month refills (Table 2).

**Table 2:** Impact of intervention on proportion of stable patients on 3-month refills: Model results using cluster summaries.

Model	Estimate interpretation	Test	Estimate	Lower CL	Upper CL
**Primary**	Difference in the mean change between intervention and control	Unpaired t-test using unweighted cluster summaries	0.15	0.02	0.29
**2**	Difference in the mean residuals of change	Unpaired t-test adjusting for average visit to HCW ratio*	0.29	-0.85	1.42
**3**	Difference in the mean residuals of change	Unpaired t-test adjusting for average number of stock-outs†	0.13	-1.01	1.28
**4**	Difference in the mean residuals of change	Unpaired-test adjusting for average visit to HCW ratio and average number of stock-outs	0.27	-0.86	1.39

*Average visit to HCW ratio is the average number of patient visits per day per healthcare worker at the facility

^†^Average number of stock-outs is average number of days per month that the stock balance for any first line drug is 0 for more than 1 day and stock data is not missing

Though significant differences in facility-level factors at baseline were not found, theoretical confounders at baseline (e.g. patient visit to health care worker ratio and average stock outs) were examined and could explain some of the differences in the primary outcome. Considering our total sample size was 16 facilities, covariate-adjusted t-tests were performed rather than regressions as considered the more robust analysis. As shown in Table 2, focusing on the significant differences in the residuals between our treatment arms, we found that the 95% confidence intervals crossed the null value of 1.0 in all three models and were thus not significant.

The ln-transformed analysis, multi-level regression model (GEE) and non-parametric tests were used to assess the robustness of the result of the primary analyses. The results of these sensitivity analyses similarly found differences between the change in three-month refills between the intervention and control group. As the inference from these findings did not vary from the primary analysis, data from these models are not shown.

### Impact of intervention on volume of clinic visits

On average, control facilities had 15 more visits per day in the endline than in the baseline, while intervention facilities had 20 fewer visits per day in endline than in baseline, which suggests that the intervention was associated with a mean difference of 35 fewer visits per day (Table 3). However, this was a non-significant result (*P* = 0.1). For the covariate-adjusted t-test, we adjusted for daily patient visit to health care worker ratio at baseline to account for varying facility size and resources.

**Table 3:** Impact of intervention on the change in daily patient visits: Model results using cluster summaries.

	Estimate interpretation	Method	Estimate	Lower CL	Upper CL
**Primary**	Mean difference between intervention and control in the change in number of daily patient visits between baseline and endline	Unpaired t-test using unweighted cluster summaries	-35.6	-79.7	8.6
**2**	Difference in the mean residuals of change	Modeling Residuals, baseline average visit to HCW ratio*	0.2	0.15	0.38

*Average visit to HCW ratio is the average number of patient visits per day per healthcare worker at the facility

### Impact of intervention on patient wait time

Patient wait time generally decreased throughout the course of the intervention at intervention sites. In comparison, the wait time for the control facilities increased throughout the course of the intervention. As a result, the change in the mean facility total wait time for intervention facilities dropped 19 minutes between baseline and endline when compared to the change of total wait time of the control facilities (95% CI: -10.2–48.5; *P* = 0.2). However, this result was non-significant. To account for the varying facility size and resources, we adjusted for average daily patient load and pharmacy staff at baseline. All results demonstrated a similar positive but non-significant trend.

### Impact of intervention on stock

The facilities that were part of the study varied greatly in size and number of visits per month, but these data suggest that stock challenges may not be as large of an issue as perceived. Stock data was analysed to identify whether intervention rollout led to stock challenges. In the intervention months of February to April, stock levels decreased, but not to below pre-intervention levels.

## Discussion

Based on the evaluation results, we consider this to be both an effective and sustainable intervention that could be expanded to the rest of Lusaka and other urban areas of Zambia. In terms of cost and sustainability, we would expect on-going savings in per patient costs if the intervention were scaled up to additional sites, primarily due to increased workforce efficiency. Direct cost savings would likely accrue due to a reduction in health care worker time as stable patients transition from twelve to four prescription refill visits each year, and health workers have time available to reinvest within the facility. Indirect cost savings could also accrue as stable patients visit facilities less frequently each year, thus allowing for nurses and pharmacists to increase annual patient volumes. This efficiency improvement could reduce the per patient cost of overhead expenditure including facility-level training, clinical and non-clinical equipment and supplies, vehicle and building maintenance and other miscellaneous administrative recurring costs such as transport and security.

Future assessments to document the sustainability and the direct pathway from three-month refills to decongestion would complement our results. While the outcomes were selected to allow for a short-term impact evaluation, the three-month window of evaluation allowed for limited measure of intervention sustainability.

Facilities were included in the randomization pool if they met the following criteria: 1) they were not already participating as a study site in another research study, and 2) they collected data with an electronic data system. These criteria narrowed the eligible pool of facilities to only 21 a small number from which to draw 16 study facilities, limiting their randomness. Further, whilst the protocol stated matched-pair analysis would be used to improve balance during randomization, matching was not kept for our primary analysis based on poor efficiency and evidence from similar RCT studies that broke the matches [21]. However, we did conduct the pair-matched analysis to confirm the findings did not vary. Patient visit times may not have been representative of the entire facility population; in order to improve the representativeness of the sampled population, visit times were measured on at least three days per facility.

## Conclusions

The UNAIDS global HIV treatment target of 90% diagnosed, 90% on treatment, and 90% virally suppressed [25] is within reach for Zambia. With over ten years of experience providing HIV services in the country, Zambia is actively addressing its implementation and service delivery challenges, some of which are most relevant to its urban areas, notably clinic congestion. Crowded HIV treatment facilities present challenges for both patients and health care providers. Larger crowds translate into longer wait times for patients and an uncomfortable waiting environment. For health care workers, more patients to see means a longer day of clinical care, sometimes requiring shorter visits per patient in order to get through the queue by the time the facility closes. Less time per patient can compromise quality.

HIV programs are increasingly moving towards more patient-friendly service delivery: visits for family consolidated into one rather than each member having a separate appointment [26]; decentralizing HIV services to rural areas [27–30]; integrating HIV services into other well-visit services such as childhood immunizations to streamline visits [31]; and community members providing one another with drug pick-up and adherence support [12, 32, 33].

The results of this trial show that, in ART facilities within urban Lusaka, district- and facility-level quality improvement activities can increase adherence to the three-month prescription refill policy outlined in the national HIV treatment guidelines [15]. A more patient-centred service delivery schedule of three-month prescription refills for stable patients is a viable step towards reducing congestion in crowded clinics, improving the patient experience, improving retention and adherence, and ultimately improving clinical outcomes. We encourage the expansion of this sustainable intervention in Zambia’s urban clinics.

## Supporting information

S1 File ART prescription refill guidelines job aide. Pharmacist job aide reminding pharmacists of the three-month ART refill policy at the time of drug dispensation.
(PDF)

S2 File Intervention cost estimates. Annual facility and district level intervention costs. Calculations of facility- and district-level intervention costs were run on an annual basis.
(DOCX)

S3 File ARV planning and quantification tool. Stock planning tool developed to quantify the expected surge in demand for drug stocks due to changes in refill practice.
(XLSX)

S4 File Initiative launch checklist for quality improvement officers. Checklist used by Quality Improvement Officers at the beginning of the intervention to ensure readiness of all clinical and pharmacy staff, and readiness of the facility’s laboratory, pharmacy, and patient filing systems.
(DOCX)

S5 File Weekly ART clinic checklist for quality improvement officers. Weekly checklist used by Quality Improvement Officers to assess drug and lab stock levels, ensure health care worker implementation of the three-month refill policy, record and troubleshoot challenges faced by the facility, and communicate those to the district level for resolution.
(DOCX)

S1 Protocol Study protocol submitted to Excellence in Research Ethics and Science (ERES), a private institutional review board in Lusaka, Zambia, in July 2014. (PDF)

S2 Protocol Study protocol amendment submitted to Excellence in Research Ethics and Science (ERES), a private institutional review board in Lusaka, Zambia, in December 2014. (PDF)

S1 Checklist CONSORT checklist of information to include when reporting a randomized trial. (DOC)
