# Supplementary material for: Quality improvement intervention to increase adherence to ART prescription policy at HIV treatment clinics in Lusaka, Zambia: A cluster randomized trial
Source: PLoS One. 2017 Apr 18;12(4):e0175534. doi: 10.1371/journal.pone.0175534 (PMC5395211; doi:10.1371/journal.pone.0175534)
Supplement: S1 File — Pharmacist job aide reminding pharmacists of the three-month ART refill policy at the time of drug dispensation. (PDF) [file pone.0175534.s001.pdf]

THE ZAMBIAN NATIONAL ART GUIDELINES RECOMMEND 3-MONTH ART REFILLS FOR STABLE, ADULT PATIENTS.

ONE MONTH REFILLS VS THREE MONTH REFILLS

One month refills

365 DOSES GIVEN PER YEAR

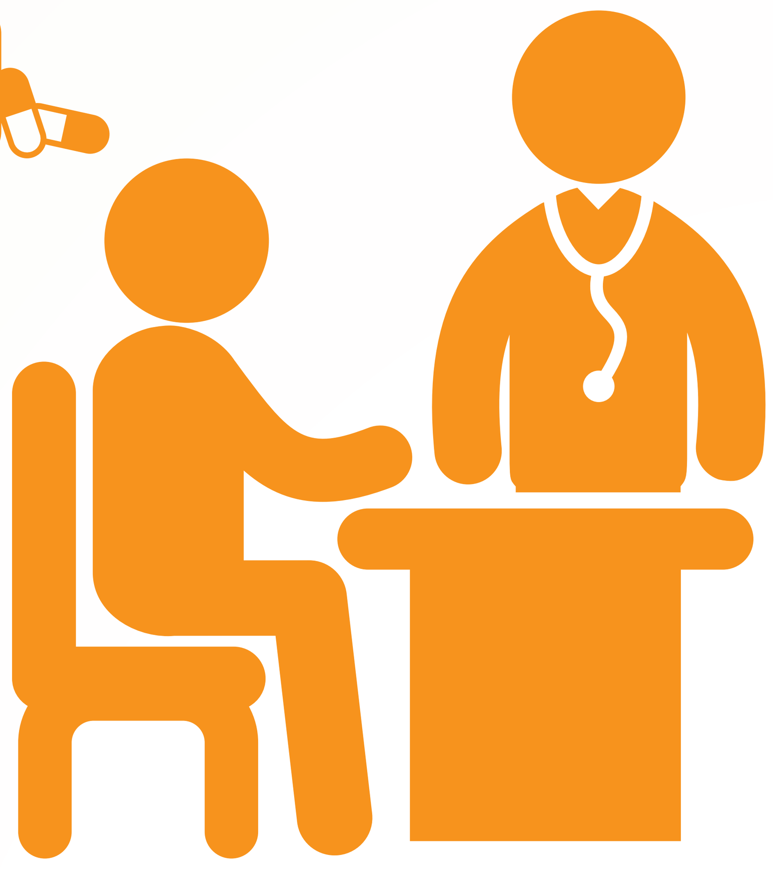

12 VISITS PER YEAR

Three month refills

365 DOSES GIVEN PER YEAR

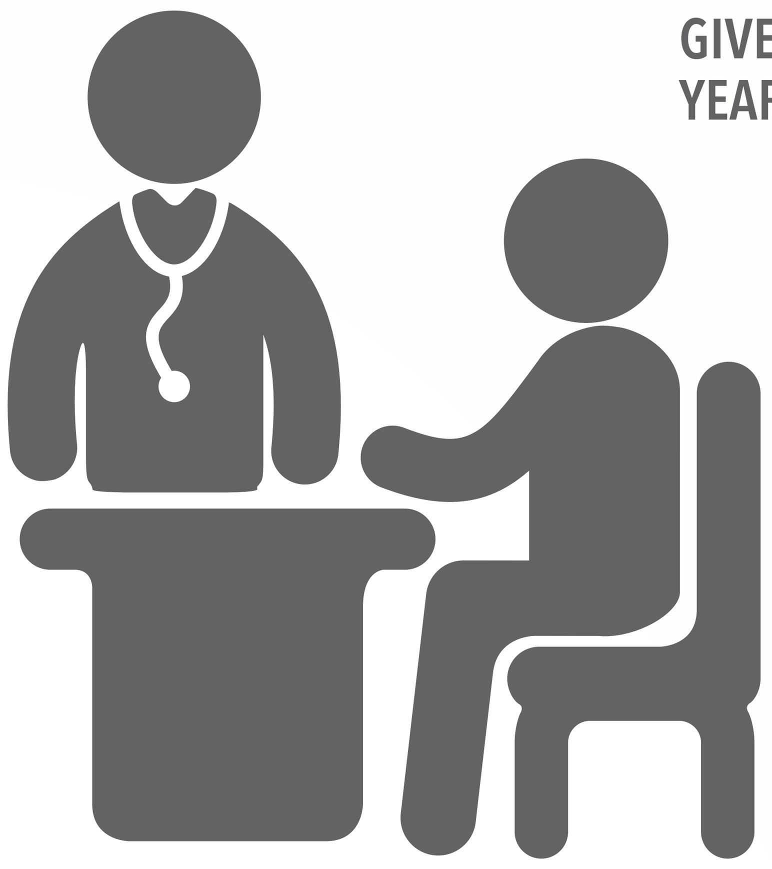

4 VISITS PER YEAR

- GIVING STABLE PATIENTS 3-MONTH REFILLS CAN:
  - I) HELP REDUCE CLINIC CONGESTION , SO YOU CAN PROVIDE MORE CARE TO THE PATIENTS THAT NEED IT
  - ii) HELP REDUCE YOUR WORKLOAD, BY REDUCING THE NUMBER OF TIMES THE SAME PATIENT COMES TO THE PHARMACY IN A YEAR
  - iii) HELP IMPROVE PATIENT SATISFACTION, BY STREAMLINING SERVICES
- 3-MONTH REFILLS SHOULD NOT CAUSE STOCKOUTS, BECAUSE PATIENTS RECEIVE THE SAME NUMBER OF PILLS OVER TIME. AT THE BEGINNING, FACILITIES MUST PLAN FOR ADDITIONAL STOCK AS PATIENTS ARE TRANSITIONED TO 3-MONTH REFILLS.

PHARMACY STEPS FOR DETERMINING REFILL LENGTH

STEP 1: Is There Sufficient Stock For At Least 5 More Clinic Days?

- **YES** → **STEP 2A:** Did The Provider Prescribe A 3-month Refill?
  - **YES** → **STEP 3A:** Provide 3-month Refill.
  - **NO** → **STEP 3B:** Does The Patient Seem To Be A Stable Patient?
    Criteria: Over 18; on current 1st line treatment regimen for at least 6 months; no opportunistic infections in last 3 months
    - **YES** → **STEP 4A:** Alert Prescriber So File Review Can Be Conducted.
    - **NO** → **STEP 4B:** Give As Prescribed
- **NO** → **STEP 2B:** Alert The Facility In- Charge And Quality Improvement Officer Immediately.

ONE ZAMBIA ONE NATION
