# Supplementary material for: Quality improvement intervention to increase adherence to ART prescription policy at HIV treatment clinics in Lusaka, Zambia: A cluster randomized trial
Source: PLoS One. 2017 Apr 18;12(4):e0175534. doi: 10.1371/journal.pone.0175534 (PMC5395211; doi:10.1371/journal.pone.0175534)
Supplement: S2 File — Annual facility and district level intervention costs. Calculations of facility- and district-level intervention costs were run on an annual basis. (DOCX) [file pone.0175534.s002.docx]

| **Annual Intervention Cost Estimates** | | | | | | |
| --- | --- | --- | --- | --- | --- | --- |
| **Exchange rate (ZMW to USD): 7.0607** | | |  |  |  |  |
|  |  | | **Cost (ZMW)** | **Frequency** | **Annual Costs (ZMW)** | **Annual Costs (USD)** |
| **Annual Costs at Facility Level** | | |  |  |  |  |
| **1** | | **Incentives** |  |  | **12,000** | **1,700** |
| 1.1 | | QI Officers Allowance | 1,000 | Month | 12,000 | 1,700 |
| **2** | | **Training Costs** |  |  | **375** | **53** |
| 2.1 | | Transport refund per participant | 175 | Year | 175 | 25 |
| 2.2 | | Hiring of Venue | 200 | Year | 200 | 28 |
| **3** | | **Operational Costs** |  |  | **27,830** | **3,941** |
| 3.1 | | Air time for QI officers & Intervention Manager | 100 | Month | 1,200 | 170 |
| 3.2 | | Transport costs | 1,000 | Month | 12,000 | 1,700 |
| 3.3 | | Facility Implementation Funds | 1,200 | Month | 14,400 | 2,039 |
| 3.4 | | Printing of Pharmacy Job aide | 150 | Year | 150 | 21 |
| 3.5 | | Printing launch checklist | 1.5 | Year | 2 | 0.2 |
| 3.6 | | Printing weekly checklist | 1.5 | Week | 78 | 11 |
|  | | **Per Facility Sub-total** |  |  | **40,205** | **5,694** |
|  | | **Number of Facilities for Scale-up** |  |  | **56** |  |
|  | | **Cost of Scale-up at All Facilities** |  |  | **2,251,452** | **318,871** |
|  | |  | **Cost (ZMW)** | **Frequency** | **Annual Costs (ZMW)** | **Annual Costs (USD)** |
| **Annual Costs at District Level** | | |  |  |  |  |
| **1** | **Incentives** | |  |  | **30,000** | **4,249** |
| 1.1 | Intervention Manager Allowance | | 2,500 | Month | 30,000 | 4,249 |
| **3** | **Operational Costs** | |  |  | **15,600** | **2,209** |
| 3.1 | Fuel refunds District staff | | 800 | Month | 9,600 | 1,360 |
| 3.2 | District Staff transport refunds | | 500 | Month | 6,000 | 849.77 |
|  | **Per District Sub-total** | |  |  | **45,600** | **6,458** |
|  | **Cost of District-Wide Scale-up** | |  |  | **2,297,052** | **325,329** |

|  |  | **Annual Costs (ZMW)** | **Annual Costs (USD)** | **% of Total Costs** |
| --- | --- | --- | --- | --- |
|  | **Cost Component Analysis** |  |  |  |
| **1** | **Incentives** | **702,000** | **99,424** | **30.6%** |
| **2** | **Training Costs** | **21,000** | **2,974** | **0.9%** |
| **3** | **Operational Costs** | **1,574,052** | **222,931** | **68.5%** |
|  | **Total** | **2,297,052** | **325,329** | **100%** |
