# Supplementary material for: Quality improvement intervention to increase adherence to ART prescription policy at HIV treatment clinics in Lusaka, Zambia: A cluster randomized trial
Source: PLoS One. 2017 Apr 18;12(4):e0175534. doi: 10.1371/journal.pone.0175534 (PMC5395211; doi:10.1371/journal.pone.0175534)
Supplement: S4 File — Checklist used by Quality Improvement Officers at the beginning of the intervention to ensure readiness of all clinical and pharmacy staff, and readiness of the facility’s laboratory, pharmacy, and patient filing systems. (DOCX) [file pone.0175534.s004.docx]

**Lusaka ART Clinic Decongestion Initiative**

**Initiative Launch Checklist for Quality Improvement Officers**

*Directions: The following checklist should be completed at the beginning of the initiative during the first QI Officer visit to each facility. The QI Officer should return on each clinic day and perform the check list again until all items are marked as “Yes”.*

Facility Name: ________________________ QI Officer Name: ________________________

Date Conducted (dd/mm/yy): ________________________

| **ART station** | **Criteria to aim for** | **Yes** | **No** |
| --- | --- | --- | --- |
| 1. **All** | 1.1 QI Officer has undertaken QI orientation training for all clinical staff (including ART providers, pharmacy staff, lab staff and adherence counsellors) |  |  |
| 1. **Drug Stock** | 2.1 Facility has undertaken quantification exercise to prepare for surge in stock as patients are transferred over to 3-month refills (See **Form 3**) |  |  |
|  | 2.2 Facility has placed “surge” drug order based on the quantification exercise |  |  |
|  | 2.3 Clinic & facility have sufficient stocks of all first line regimes to provide all stable patients with a 3-month refill |  |  |
|  | 2.4 Stock request intervals are documented appropriately and planned given storage restrictions |  |  |
| 1. **Laboratory Stock** | 3.1 Facility has undertaken quantification exercise to ensure adequate laboratory supply stocks are in place |  |  |
|  | 3.2 Facility has appropriate stock in place for at least 5 more clinic days |  |  |
|  | 3.3 Request intervals are documented appropriately planned given storage restrictions |  |  |
| 1. **Pharmacy** | 4.1 Pharmacy job aid is visible (See **Form 4**) |  |  |
|  | 4.2 Pharmacist has been trained in and agrees to follow steps described on job aid |  |  |
| 1. **Clinician** | 5.1 Clinicians are aware of policy and guidance about 3-month refills |  |  |
| 1. **File storage** | 6.1 Patient files are stored according to MOH guidelines |  |  |
