# Supplementary material for: Quality improvement intervention to increase adherence to ART prescription policy at HIV treatment clinics in Lusaka, Zambia: A cluster randomized trial
Source: PLoS One. 2017 Apr 18;12(4):e0175534. doi: 10.1371/journal.pone.0175534 (PMC5395211; doi:10.1371/journal.pone.0175534)
Supplement: S5 File — Weekly checklist used by Quality Improvement Officers to assess drug and lab stock levels, ensure health care worker implementation of the three-month refill policy, record and troubleshoot challenges faced by the facility, and communicate those to the district level for resolution. (DOCX) [file pone.0175534.s005.docx]

**Lusaka ART Clinic Decongestion Initiative**

**Weekly ART Clinic Checklist**

*Directions: The following checklist should be completed at least once a week during QI Officer visits to facilities using the data sources specified below.*

**Facility Name: ________________________ QI Officer Name: ________________________**

**Date Conducted (dd/mm/yy): ________________________**

***Part 1. Pharmacy observations***

| **Indicator** | **Data source** | ***(1) Yes, independently without coaching*** | ***(2) Yes, but only after coaching*** | ***(3) No, not even after coaching*** |
| --- | --- | --- | --- | --- |
| Number of patients for whom pharmacist is accurately following steps of job aid (See **Form 4**) | Observe pharmacist providing **10** ART prescriptions and coach them when not following job aid | out of 10 | out of 10 | out of 10 |

| **In cases where the pharmacist does not follow the steps of the job aid even with coach (response #3 above), explain the situation:** |
| --- |
|  |
|  |
|  |
|  |
|  |

***Part 2. Patient record reviews***

| **Indicator** | **Data source** | ***(1) Number that prescribed a 3-month refill*** | ***(2) Number that were not prescribed a 3-month refill*** |
| --- | --- | --- | --- |
| Number of stable patients that have been prescribed 3-month refills | Review **20** randomly selected patient files for patients visiting clinic in last week (continue selecting files until you get 20 stable adults) | out of 20 | out of 20 |

| **In cases where 3-month refills were not provided, review the cases with clinical staff. Below, describe reasons that they give for the situation:** |
| --- |
|  |
|  |
|  |
|  |
|  |

***Part 3. Stock inventory check***

| **Indicator** | **Data source** | ***(1) Yes*** | ***(2) No, but order has been placed*** | ***(3) No, and not order has been placed*** |
| --- | --- | --- | --- | --- |
| Does the facility have adequate supplies of the **standard first line regimen** available for at least 5 more clinic days  *(In most clinics this is[TDF + FTC + EFV] or TDF + 3TC + EFV] but this may vary across clinics)* | Observe inventory and ask inventory manager |  |  |  |
| Adequate supply of **LAB TESTING COMMODITIES** for at least 5 more clinic days | Observe inventory and ask inventory manager |  |  |  |

| **For the following table, use facility inventory records and consultations with the inventory manager to quantify the current stocks of first-line drugs. (include appropriate unit)** | | |
| --- | --- | --- |
| **First-Line ARTs** | **Quantity Available in**  **ART Clinic** | **Quantity Available in Health Facility** |
| TDF + FTC + EFV |  |  |
| TDF + 3TC + EFV |  |  |
| TDF + FTC |  |  |
| TDF + 3TC |  |  |
| NVP (NEVIRAPINE) |  |  |
| ABC (ABACAVIR) |  |  |
| 3TC (LAMIVUDINE) |  |  |
| EFV (EFAVIRENZ) |  |  |
