# Supplementary material for: Quality improvement intervention to increase adherence to ART prescription policy at HIV treatment clinics in Lusaka, Zambia: A cluster randomized trial
Source: PLoS One. 2017 Apr 18;12(4):e0175534. doi: 10.1371/journal.pone.0175534 (PMC5395211; doi:10.1371/journal.pone.0175534)
Supplement: S1 Protocol — (PDF) [file pone.0175534.s006.pdf]

Clinton Health Access Initiative
Plot 175 Kudu Road
Kabulonga, Lusaka, Zambia
PO Box 51071 Ridgeway, Lusaka

RE: A study to increase the proportion of stable patients on antiretroviral therapy receiving 3-month refills

July 30, 2014

Dear ERES Ethics Committee Secretary,

Please accept our protocol for a study looking at interventions to improve clinic efficiency and supply chain systems to ERES CONVERGE for your review. Please contact me with any questions or requests for additional information.

Yours sincerely,

Sarah Moberley

Dr Sarah Moberley
Co-Principle Investigator
Clinton Health Access Initiative

STUDY PROTOCOL

Version 1.0, 31 July 2014

**Study Title:**	Determining the effectiveness of improved clinic efficiency and supply chain quality on the proportion of stable patients on 3-month refills at 4 months post intervention: A pair-matched, randomised, difference in difference study
**Abbreviated Title:**	Impact of improved clinic efficiency and supply chain quality on antiretroviral therapy refill length
**Trial Registration #:**	To be advised
**Principal Investigators:**	Dr Albert Mwango, BHB, BMBS, MPH<br>Dr Sarah Moberley, BN, MPH, PhD
**Chief Investigators:**	Dr Marta Prescott, MPH, PhD<br>Hamsa Subramaniam, BA, MPH<br>Elizabeth McCarthy, BA, MPH<br>Margaret Lippitt, BA, MPH
**Associate Investigators:**	Felton Mpasela<br>Benjamin Chibuye
**Administering Institution:**	Clinton Health Access Initiative
**Dates**	August 2014 to May 2015

1 INVESTIGATOR AGREEMENT

I agree:

- To assume responsibility for the proper conduct of the study;
- To conduct the study in compliance with this protocol, with any future protocol amendments and with any study conduct procedures;
- To ensure that all persons involved with this study are adequately informed about the study-related duties and functions as described in the protocol;
- Not to implement any changes to the protocol without prior review and approval from the IRB approving the protocol;
- That I am aware of, and will comply with "Good Epidemiological Practice" (GEP);
- That I, and any persons employed on this project, will provide up to date curriculum vitaes and any declaration of financial and ownership interests in this project.

Investigator name: ____

Date:_________________

2 STUDY SYNOPSIS

Facility congestion is a significant concern in Zambia. With demand for treatment greater than the available capacity of health infrastructure, the MOH and other stakeholders are concerned about the effect that congestion within facilities may have on patient retention in Antiretroviral therapy care, particularly in urban areas.

We intend to conduct a rapid impact evaluation as part of the DFID funded Demand-Driven Evaluations for Decisions project. This proposal has been designed in response to the question demanded by the Ministry of Health in Zambia 'How to decongest busy ART clinics in Lusaka.' We seek to generate reliable impact evidence that meets the Ministry's needs and is used to catalyze implementation of cost effective action.

This proposal outlines a study with two components. The first being an intensive assessment period during which time, critical process failures are identified that prevent the provision of 3-month ART refills and the design of an intervention that improves clinic efficiency and improves the supply of ART commodities. The second component is a matched, randomised difference in difference study that will determine the effectiveness of the intervention.

We hypothesise that improvements to clinic efficiency and improving the supply of ART stocks will result in a higher proportion of patients receiving 3 month refills, which in turn will reduce the frequency of patients visiting the ART clinic by up to a third and the congestion level of the clinic will improve.

Table of Contents

1 INVESTIGATOR AGREEMENT 2
2 STUDY SYNOPSIS 3
3 GENERAL INFORMATION 6
3.1 Protocol full title 6
3.2 Principal investigator 6
3.3 Person(s) authorised to sign the protocol amendments 6
3.4 Investigator(s) responsible for conducting study 6
3.5 Other institutions involved 7
4 AIMS 7
4.1 Objectives 7
5 BACKGROUND AND RATIONALE 8
5.1 Introduction 8
5.2 ART Clinic Congestion 8
5.3 ART refill length 9
5.4 Program description and theory of change 9
5.5 Rationale for Study 10
6 RESEARCH PLAN 10
6.1 Study design 10
6.1.1 Study population 11
6.1.2 Sampling methodology 11
6.1.3 Study schema 11
6.2 Eligibility for recruitment 13
6.1.4 Inclusion criteria 13
6.1.5 Exclusion criteria 13
6.3 Study procedures 13
6.3.1 Clinic efficiency and supply chain assessment 13
6.3.2 Intervention design 14
6.3.3 Assessment of the impact of the intervention 14
6.3.4 Ethical considerations 14

6.4 Sample size calculation ... 15
6.5 Primary and secondary analyses ... 16
6.5.1 Secondary analyses ... 16
6.5.2 Stopping Rules ... 17
6.5.3 Study adaptation ... 17
6.5.4 Dissemination ... 17
7 ADMINISTRATIVE ASPECTS ... 17
7.1 Monitoring ... 17
7.2 Recording of data ... 17
7.3 Data quality control ... 18
7.4 Confidentiality ... 18
8 REFERENCES ... 19
ANNEX 1 ART Register Data Collection Form ... 21
ANNEX 2 Baseline Facility Data Collection Form ... 22
ANNEX 3 Patient Flow Data Collection Form ... 24
ANNEX 4 Pharmacy Register Data Collection Form ... 25
ANNEX 5 ART Stock Out Data Collection Form ... 26
ANNEX 6 Key Informant Interview Data Collection Form ... 27
ANNEX 7 Patient Exit Interview Data Collection Form ... 32
ANNEX 8 Key Informant Consent Form ... 38
ANNEX 9 Patient Exit Interview Consent Form ... 39
ANNEX 10 Key Informant Participant Information ... 40
ANNEX 11 Patient Exit Interview Participant Information ... 42
ANNEX 12 Principle Investigator CV Dr Mwango ... 46
ANNEX 13 Principle Investigator CV Dr Moberley ... 51

3 GENERAL INFORMATION

3.1 Protocol full title

Determining the impact of clinic efficiency and supply chain quality improvement interventions on antiretroviral therapy refill length: A matched, randomised difference in difference study

3.2 Principal investigators

Dr Albert Mwango, National ART Coordinator, Zambia Ministry of Health

Dr Sarah Moberley, Senior Technical Advisor, Clinton Health Access Initiative

3.3 Person(s) authorised to sign the protocol amendments

Sarah Moberley and Elizabeth McCarthy, Clinton Health Access Initiative

3.4 Investigator(s) responsible for conducting study

Sarah Moberley	Senior Technical Advisor, Clinton Health Access Initiative
Elizabeth McCarthy	Director, Applied Analytics Team, Clinton Health Access Initiative
Marta Prescott	Senior Technical Advisor, Applied Analytics Team, Clinton Health Access Initiative
Hamsa Subramaniam	Research Associate, Applied Analytics Team, Clinton Health Access Initiative
Margaret Lippitt	Research Associate, Applied Analytics Team, Clinton Health Access Initiative
Felton Mpasela	Program Associate, Clinton Health Access Initiative
Benjamin Chibuye	Program Manager, Clinton Health Access Initiative

3.5 *Other institutions involved*

The following study sites in Lusaka District are proposed to participate pending eligibility assessment:

Bauleni Urban Health Center,Bwafwano Community Urban Health Center, Chazanga Urban Health Center, Chelstone Urban Health Center, Chilenje Urban Health Center, George Urban Health Center, Kabwata Urban Health Center, Kalingalinga Urban Health Center, Kamwala Urban Health Center, Kara Clinic, Makeni Urban Health Center, Matero Main Urban Health Center, Matero Referral Urban Health Center, Mtendere Urban Health Center, Ng'ombe Urban Health Center, Lusaka Railway Urban Health Center, SOS Medical Center, State Lodge Urban Health Center, University of Zambia (UNZA) Health Center.

4 AIMS

The primary aim is to determine the impact of clinic efficiency and supply chain quality interventions on the proportion of stable patients on 3-month refills at 4 months post intervention.

The secondary aims are as follows:

a) Estimate the effectiveness of improved clinic efficiency and supply chain quality on other factors associated with antiretroviral therapy (ART) outcomes at 4 months post intervention such as patient wait times and patient satisfaction

b) Assess the factors that enable the intervention to be successful, including qualitative process measures

- Patient satisfaction and perceptions of quality of care
- Patient experiences with transition to multi-month refills
- Patient and clinician perceptions on reliability of supply chain
- Clinician perspectives on barriers or challenges to providing multi-month refills
- Compliance to quality improvement standard operating procedures

4.1 *Objectives*

The primary objectives are:

1. Critically assess the failures and challenges related to clinic efficiency and supply chain and their role in preventing the provision of 3-month ART refills.
2. Plan and execute a set of interventions based on the assessment of failures and challenges related to facility congestion.
3. After implementation of the interventions, to determine their impact on the proportion of stable ART patients on 3-month refills.

5 BACKGROUND AND RATIONALE

5.1 Introduction

Demand-Driven Evaluations for Decisions (3DE) is a three-year program funded by DFID. The program supports rigorous impact evaluation in areas of utmost importance to the Ministry of Health (MOH). It seeks to generate reliable impact evidence that meets the Ministry's needs and is used to catalyze implementation of cost effective action. This protocol is a 3DE Evaluation, and as such, this research question was demanded by the MOH.

5.2 ART Clinic Congestion

In June 2013, the WHO released guidelines that increase the number of people eligible for antiretroviral therapy (ART) from close to 17 million to more than 26 million people in low- and middle-income countries.[1] This decision reflects significant evidence that increasing treatment coverage and access to high-impact prevention interventions will be a crucial next step in turning the tide of the AIDS epidemic. However, despite significant treatment scale-up over the past decade, countries in Sub Saharan Africa are far from achieving the target of universal access for all those in need and will face numerous challenges in scaling up HIV treatment. In particular, the infrastructure, health system and human resources required to properly provide and monitor treatment are extensive, and current systems have limited capacity to support scaled-up ART programs.[1,2,3] With the roll-out of strategies to improve access for treatment such as Option B+, existing ART facilities are crowded and burdened, and with the continued scale-up of the provision of ART, they will continue to become increasingly congested.

---

[1] Eligibility under the 2010 Guidelines was originally estimated at 15 M and has since been revised based on changes in the epidemic and modeling. Global Update on HIV Treatment 2013. WHO Report.

Facility congestion is a concern in Zambia where only 450 accredited health facilities provided treatment to approximately 566,000 patients in 2013.[4] Based on the UNAIDS Gap Report, only about one third of HIV positive patients are accessing treatment, suggesting that there are a total of over one million patients[5] are estimated to be in need of ART in Zambia. Although the MOH is building 650 more ART accredited health facilities to meet the demand, it is essential to identify opportunities for increased efficiencies in order to accommodate these patients. With demand for treatment greater than the capacity within Zambia, the MOH and stakeholders are concerned about the effect that congestion within facilities may have on patient retention in ART care, particularly in urban areas.

Although there is an absence of published literature regarding the implication of congestion on patient retention in ART care, there is evidence to suggest that congestion may affect patient flow and wait times.[6] Moreover, experienced health staff within Zambia have reported that long wait times lead to decreased ART retention, and subsequently, poor treatment outcomes.

5.3 ART refill length

One factor affecting facility congestion is the proportion of stable ART patients receiving 3-month refill prescriptions. With longer prescription refill length, as compared to the frequently dispensed 1- or 2-month prescriptions, stable patients are able to space out their facility visits, and thus, reduce the number of people within the facility at any given time. The Zambian ART Guidelines recommend that stable ART patients be provided with prescriptions for up to 3 months at a time,[7] but few patients in Zambia receive such prescriptions.

To assess refill patterns within Zambian facilities, our team conducted assessments at five ART clinics in Lusaka. The majority of patients received their ART refill for one month or less (51%), 26% of patients received a 2-month refill and 23% received a 3-month refill. The decision on the ideal refill length is often decided by a team (including adherence counsellors and clinicians), however the pharmacist made the final decision based their knowledge of drug stock levels. The In-Charge of facilities reported that unreliable stocks of ARTs were the main barrier to the provision of 3-month refills for stable patients.

5.4 Program description and theory of change

With the aim of improving facility-level congestion and based on the results from the preliminary assessment, interventions are being proposed to address facility specific issues centered on clinic efficiency and supply chain quality. A potential theory of change is listed below:

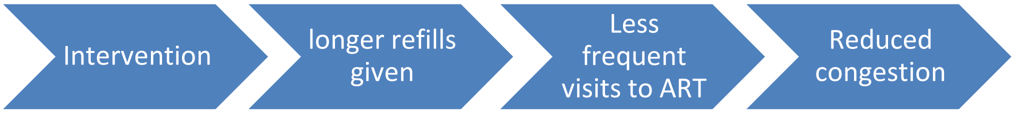

Improved clinical efficiency and supply systems including SOPs

Efficiency and supply procedures lead to an increase in the % stable patients on 3-month refills

Longer refills mean patients visit ART clinic less often

Less frequent appointment leads to fewer patients attending on any given day

5.5 *Rationale for Study*

The purpose of this work is to outline the impact of the potential intervention for ART clinic efficiency and supply chain quality on facility congestion as measured by the proportion of stable ART patients on 3-month ART prescriptions. We aim to develop and test clinical efficiency and supply chain quality interventions that can be utilized in public, urban facilities throughout Zambia, in order to facilitate the process of scaling up access to ART in Zambia.

6 RESEARCH PLAN

6.1 *Study design*

We will conduct a cluster randomised, pair-matched, difference in difference study to assess the impact of the intervention on the proportion of 3-month refill scripts. To assure that the two arms (i.e. the control and the intervention arms) are as comparable as possible, facilities will be matched into pairs based on important factors associated with the primary outcome including the proportion of patients on 3-month refills at baseline, the size of the facility, staff-to-patient ratio (active ART patients) and distance from the Medical Stores Limited (MSL). After pair matching, one facility from each pair will be

randomly assigned to undergo the intervention with a 3-month period of assessment and baseline data collection, followed by a 4-month period of the intervention. To assess facility-level outcomes data will be collected in various formats including patient records, patient exit interviews, provider interviews and other relevant key informant interviews.

6.1.1 Study population

For this work, the study population will be ART facilities within the Lusaka region of Zambia. Patient study population are those who attend one of 16 selected ART facilities within Lusaka, Zambia from August 2014 to March 2015.

6.1.2 Sampling methodology

There are four types of sampling methods based on the method of data collection: facility, patient records, patient exit interviews, and key informant interviews (including health providers and District and MSL staff responsible for ART supply)

- Facilities will be purposefully selected based on the proximity within the Lusaka catchment area and to assure specific key facilities are within the sample for the purposes of policy decisions. Within each facility, one key informant staff member will be interviewed to provide facility-level information.
- Adult (aged 18 or more) patient records will be collected for all eligible patients who receive care at the facility within the time period (provided they meet our eligibility criteria).
- For patient exit interviews, patients will be randomly selected throughout certain days to assure there is a representative sample of the ART facility patients. To this end, every 10th patient to leave the facility will be asked to participate in an exit interview. If this patient does not wish to participate, the immediate next participant will be approached.
- For key informant interviews, clinicians (including doctors, clinical officers, nurses and pharmacists) will be purposefully selected to provide a range of perspectives and experiences.

6.1.3 Study schema

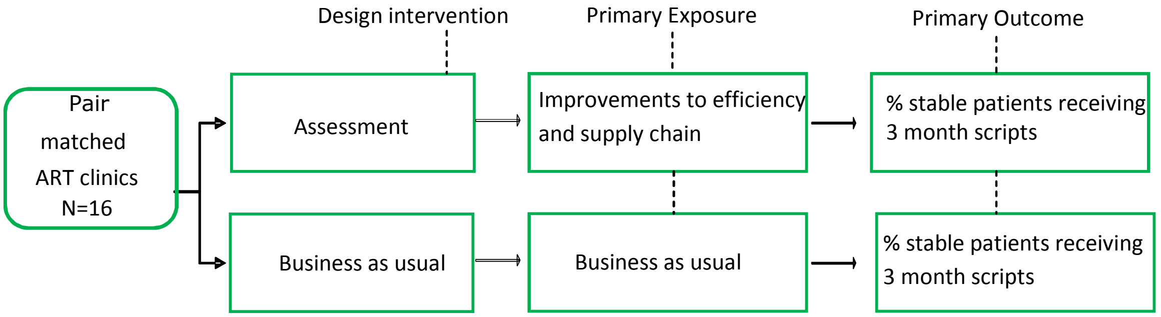

Primary Outcome

The primary outcome is the proportion of stable ART patients receiving 3-month refills, defined as:

> The number of stable patients given a 3-month refill in the previous month divided by the total number of stable patients seen in the last month.

Stable patients are defined as:

> A patient active in ART care for more than 6 months and on first-line treatment as outlined in the Zambia Consolidated Guidelines[7] (includes the following combinations (**1**) TDF + XTC + EFV, (**2**) TDF + XTC + LPV-r, (**3**) TDF + XTC + ATV-r, (**4**) TDF + XTC + NVP+, (**5**) AZT + 3TC + LPV-r, (**6**) ABC + 3TC + LPV-r, (**7**) ABC + 3TC + EFV).

Secondary outcomes include:

- Patient wait times based on the daily average wait time from entry into the clinic to medication delivery
- Compliance to quality improvement standard operating procedures
- Patient satisfaction and perceptions of quality of care
- Patient experiences with transition to multi-month refills
- Provider perspectives on process of implementing clinical efficiency and supply chain interviews and on barriers or challenges to providing multi-month refills
- Patient and provider perceptions on reliability of supply chain

6.2 Eligibility for recruitment

6.1.4 Inclusion criteria

Facilities that have agreed to participate and have the potential to increase the proportion of stable patients on 3-month refills by 20% (currently have less than 80% of stable patients on 3 month refills).

Adult ART patients aged 18 years and over will be eligible for the exit interview.

6.1.5 Exclusion criteria

Facilities will be excluded if they are currently participating in research that may interfere with our primary or secondary outcomes.

6.3 Study procedures

6.3.1 Clinic efficiency and supply chain assessment

1. The first component of the study will be a detailed assessment of failures in clinic efficiency and supply chain. This will include, but not be limited to:
   - Patient flow assessment will be measured in each site over 2 days recording the patient wait times at each component of the clinic.
   - Patient exit interviews to receive information on their experience; what they think works well at the clinic and what does not work well.
   - Data collection at the pharmacy dispensing site to identify rationale for refill interval length.
   - Data from the ART register that identifies stable patients will be linked to the pharmacy dispensing data in order to determine the baseline proportion of stable patients receiving 3 month refills.
   - An assessment of supply chain reports, requisition forms and the flow of supply related reports and commodities.
   - Key informant interviews of facility, district and MSL staff to determine each member's experiences and cited challenges in the provision of ART care and 3-month refills in particular.

- An assessment of any available quality improvement checklist and standard operating procedures related to dispensing ARTs.

Data collection forms are attached as Annex 1 to 7.

6.3.2 Intervention design

The design of the intervention will be informed by the above assessment. In particular, critical process failures will be identified and an intervention designed to resolve the issue. A stakeholder meeting will take place and involve all relevant parties including Facility in-charges, District Pharmacists and ART Coordinators, MSL staff responsible for ART supply, and collaborators such as CIDRZ. The purpose of this stakeholder meeting is to ensure that there is buy-in and ownership of the intervention from all relevant stakeholders.

6.3.3 Assessment of the impact of the intervention

Based on the randomized pair-matched study design, ongoing assessments will measure process and outcome indicators (outlined below).

6.3.4 Ethical considerations

Informed consent will be sought from each key informant or patient exit interview participant in accordance with IRB approval following an explanation of the study using a Plain Language Statement approved by the IRB. Study staff will clearly explain the rights of potential participants to decline to participate at any point without any negative consequence.

Both consent forms and patient information sheets are attached to this application as Annex 8 and 9 respectively. A minimal dataset summarising the outcomes from those approached for the Plain Language Statement will be recorded through a Screening Log.

Participant questionnaires approved by the IRB in electronic or paper format will be used to document patient wait times, information relating to the barriers/influences of being retained in ART care, patient and facility staff identified challenges regarding the provision of longer refills, and some socio-demographic indicators.

This study will be conducted according to Good Epidemiological Practises.[8] Staff will be required to have undergone training in ethics and the management of study records according to Standard Operating Procedures.

The risks of this study relate only to access of identifiable information. As outlined above the study team will go to great lengths to ensure patient confidentiality is maintained. All staff will have been trained and committed to maintain confidentiality. Only unidentifiable records will be stored in a password protected database and any original Case Report Forms will be stored in a locked filing cabinet within a locked room at the Clinton Health Access Initiative Office in Lusaka.

The benefits of the study will be for improved efficiency measures being rolled out across all sites should the MOH decide that the intervention was successful.

No compensation will be provided to participants of surveys other than refreshments.

6.4 Sample size calculation

For the pair-matched, cluster-randomized design, the sample size of 16 facilities (estimated 3845 patients per facility with a 20% potential for missing records – an estimate of 3076 per facility)[9] over the 4-month recruitment period will have a power of 80% (α=0.05) to detect difference of ≥14% for the change in the proportion of patients receiving 3-month refills in the intervention group against the change in the control group (Hayes & Bennett, 1999). The sample size calculation was performed according to the following equation for the comparison of two proportions:

$$c = 2 + \left(z_{\frac{\alpha}{2}} + z_{\beta}\right)^2 \frac{[\pi_0(1-\pi_0) + \pi_1(1-\pi_1) + (1+(m-1)\rho)]}{m(\pi_0-\pi_1)^2}$$

The equation was calculated according to the following parameters:

Parameter	Value
Alpha (α)	0.05
Power (β)	0.8
Number of patients per cluster (m)	3076
Estimated change in proportion of 3-month refills (π_0)	13%
Estimated change in proportion	1%

of 3-month refills in control (π_0)	
Rho	0.1
J (total, treatment and control groups)	16

6.5 *Primary and secondary analyses*

Firstly, to assess the ability of the matching to produce comparable groups, the characteristics of the matching factors will be compared between the control and intervention group at baseline. Secondly, to assess the presence of other potential confounders, facility-level factors such as the age distribution of the patients or proportion of the population female will be compared between the two groups. Bivariable comparisons will also compare how these potential confounders are associated with the primary outcome. After examining the success of the matching and the existence of other potential confounders, the remaining analyses will continue with matched analytical methods.

The primary analysis will be a matched comparison of the difference between the intervention and non-intervention facilities in the change of the proportion of eligible patients on 3-month refills overtime (from baseline – endpoint) adjusted for pair-matching and other relevant indicators. The denominator will include all stable patients active in care at the endline. To this end, for bivariable comparisons, we will run McNemar's matched methods to compare the difference between the proportions between the matched pairs. For further adjustments for potential confounders, conditional multivariable models will be run to examine the difference in the proportion of increased 3-month refills between the intervention and the non-intervention groups.

6.5.1 Secondary analyses

Secondary analyses will consider the impact of the intervention by comparing mean and the variability of patient wait times and score of satisfaction between the intervention and control sites. We will run similar bivariable comparisons to examine how these factors may be associated with potential confounders. Then, paired t-tests will be used to examine the mean differences between the two groups for the variable of patient wait times and other secondary outcomes. Conditional multivariable analyses will be used to account for matched analyses as well as potential confounders. In addition, we will

describe compliance to the intervention and qualitative assessment on process successes and failures as well as patient and staff satisfaction.

6.5.2 Stopping Rules

Midline analyses will be conducted and the study will cease should there be evidence of a beneficial effect of the intervention of a least a 20 percent difference between the intervention and control sites.

6.5.3 Study adaptation

During the midline analyses, we will determine whether any revisions are required to the intervention based on the experience of the first two months.

6.5.4 Dissemination

The work from this impact evaluation will be shared directly with the MOH and stakeholders to inform the potential scale up of the proposed intervention. Additionally, the results from this work will be shared with community audiences who participated in the work by presenting findings to the facilities and all key stakeholders where appropriate.

7 ADMINISTRATIVE ASPECTS

7.1 Monitoring

Study monitoring will be the responsibility of the Study Coordinator, Felton Mpasela of the Clinton Health Access Initiative, and will be conducted according to standard operating procedures.

7.2 Recording of data

Source data will be collected in study workbooks, either in paper format or in electronic form with direct data entry utilising tablets. All data will be entered into a secure electronic database. The Case Report Form (CRF) will be an electronic data file compromised of core data fields. Data will be entered directly into electronic CRFs via a secure web interface. All entries on the CRF must be backed up by source data, unless there is a note to file specifying a deviation from this requirement for a specific purpose.

Workbooks and source data must be kept in order and up-to-date so that they always reflect the latest observations on the participants enrolled in the study. Entries into workbooks must be legible and any changes or corrections to the workbook should not obscure the original entry. Handwritten entries are to be done using a non-soluble/non-smudging ball point pen. Errors are to be corrected by passing a single line through the error and accompanied by the researcher’s initials and date. White-out fluid is not to be used under any circumstance. Signature logs of all study staff are to be retained in the Central Investigator files.

7.3 Data quality control

Following completion of each component of the CRF, the data will be checked for consistency, logic and range, either through the process of direct data entry into an eCRF or through analyses of the study database. Queries will be generated for spurious data and clarification sought in writing from the Principle Investigator or delegate. Data query forms will then be forwarded back to the staff responsible for database amendment. If necessary, source documents may need to be accessed to correct data errors.

7.4 Confidentiality

All identifiable information on study subjects will be retained in password protected files and locked cabinets at study sites. Access to this information will only be provided to immediate study staff who have signed confidentiality agreements. No identifying information will be included in study reports.

8 REFERENCES

1. Kober K, Van Damme W. Scaling up access to antiretroviral treatment in southern Africa: who will do the job? Lancet. 2004;364(9428):103–107.
2. Van Damme W, Kober K, Laga M. The real challenges for scaling up ART in sub-Saharan Africa. AIDS.2006;20:653–656
3. Jaffe HW. Universal access to HIV/AIDS treatment: promise and problems. JAMA. 2008;300:573–575.
4. ART report, Zambian Ministry of Health, Quarter 3 of 2013.
5. UNAIDS. The Gap Report. 2014. http://www.unaids.org/en/media/unaids/contentassets/documents/unaidspublication/2014/UNAIDS_Gap_report_en.pdf
6. Wanyenze R, Wanger G, Alamo S, Amanyire G, Ouma J, Kwarisima D, Sunday P, Wabwire-Mangen F, Kamya M. Evaluation of the Efficiency of Patient Flow at Three HIV Clinics in Uganda. AIDS Patient Care STDS. Jul 2010; 24(7): 441–446.
7. Zambian Ministry of Health and the Ministry of Community Development Mother and Child Health. Zambia Consolidated Guidelines for the Treatment and Prevention of HIV Infection. February 2014.
8. International Epidemiological Association, Guidelines for Proper Conduct in Epidemiological Research, November 2007, http://ieaweb.org/good-epidemiological-practice-gep/, accessed 30th July, 2014.
9. Scott CA, Iyer HS, McCoy K, Moyo C, Long L, Larson BA1, Rosen S. Retention in care, resource utilization, and costs for adults receiving antiretroviral therapy in Zambia: a retrospective cohort study. BMC Public Health. 2014 Mar 31;14:296. doi: 10.1186/1471-2458-14-296.

							FACILITY ID

ART Register Data Collection Form

VERIFICATION AND FACILITY INFORMATION

Data is complete and accurate	**Name**	**Signature**	**Date (dd/mm/yyyy)**
**2.1 Enumerator**			/ / 2014
**2.2 Supervisor**			/ / 2014
**2.3 Name of facility**			

ELIGIBILITY: ALL PATIENTS ON 1ST LINE TREATMENT, 18YEARS OLD OR OVER WHO MADE AT LEAST ONE APPOINTMENT DURING THE PREVIOUS 3 MONTHS

PATIENT INFORMATION

2.4 Patient's OPD ID number	2.5 Sex (M/F)	2.6 ART start date* (dd/mm/yyyy)	2.7 Date of Birth	2.9 Date of most recent appointment (dd/mm/yyyy)	2.10 Date of next appointment (dd/mm/yyyy)	2.11 Name of digital photograph files	2.12 Notes or comments
	M F						
	M F						
	M F						
	M F						
	M F						
	M F						

*If patient has been lost to care and then re-initiated, the date required is the most recent initiation of ART

FACILITY ID								

Data is complete and accurate	**Name**	**Signature**	**Date (dd/mm/yyyy)**
**Enumerator**			/ / 2014
**Supervisor**			/ / 2014
**Name of facility**			

FACILITY INFORMATION

NO.	QUESTION	RESPONSE CODE	SKIP
FC1	HOW MANY REGISTERED NURSES?	Number .... ___ ___<br>Refused .... 97<br>Don't know 98	
FC2	HOW MANY ENROLLED NURSES?	Number .... ___ ___<br>Refused .... 97<br>Don't know 98	
FC3	HOW MANY CLINICAL OFFICERS?	Number .... ___ ___<br>Refused .... 97<br>Don't know 98	
FC4	*HOW MANY DOCTORS?*	Number .... ___ ___<br>Refused .... 97<br>Don't know 98	
FC5	HOW MANY PHARMACISTS?	Number .... ___ ___<br>Refused .... 97<br>Don't know 98	
FC6	HOW MANY CLERKS?	Number .... ___ ___<br>Refused .... 97<br>Don't know 98	

FC7	HOW MANY DAYS IS THE ART CLINIC OPEN IN ONE WEEK?	Number .... ___ ___<br>Refused .... 97<br>Don't know 98	
FC8	WHAT IS THE FACILITY POPULATION CATCHMENT?	Number .... ___ ___<br>Refused .... 97<br>Don't know 98	
FC9	HOW MANY ACTIVE PATIENTS ATTEND THIS FACILITY?	Number .... ___ ___<br>Refused .... 97<br>Don't know 98	
FC10	DO SOME PATIENT GROUPS ATTEND ON SPECIFIC ART CLINIC DAYS?	No .... 1<br>Yes .... 2<br>Refused .... 97<br>Don't know 98	
FC11	ARE SOME ART CLINIC DAYS BUSIER THAN OTHERS?	No .... 1<br>Yes .... 2<br>Refused .... 97<br>Don't know 98	
FC12	WHAT ARE THE OPERATING HOURS OF THE ART CLINIC?	Number .... ___ ___<br>Refused .... 97<br>Don't know 98	
FC13	DOES THE CLINIC CONDUCT ANY SPECIAL ACTIVITES TO RETAIN PATIENTS IN CARE OR TRACE PATIENTS LOST TO FOLLOW-UP?	No 1	
		Outreach.. 2	
		SMS reminders 3	
		Other:<br>Specify_____________________...............4	

FACILITY ID

DAILY PATIENT INFORMTION/CLINIC FLOW

1<br>ID	2<br>ARRIVAL TIME OF PATIENT		3<br>REGISTRATION TIME		4<br>TIME AT TRIAGE		4<br>CLINICAL ASSESSMENT TIME		4<br>ART DISPENSED TIME	
Line		HRS		HRS		HRS		HRS		HRS
1										
2										
3										
4										
5										
6										
7										
8										
9										
10										

Data is complete and accurate	**Name**	**Signature**	**Date (dd/mm/yyyy)**
**Enumerator**			/ / 2014
**Supervisor**			/ / 2014
**Name of facility**			

Facility ID | | | | | | | |

Pharmacy Register Data Collection Form

VERIFICATION AND FACILITY INFORMATION

Data is complete and accurate	**Name**	**Signature**	**Date (dd/mm/yyyy)**
**2.1 Enumerator**			/ / 2014
**2.2 Supervisor**			/ / 2014
**2.3 Name of facility**			

ELIGIBILITY: ALL PATIENTS ON 1ST LINE TREATMENT, 18YEARS OLD OR OVER WHO MADE AT LEAST ONE APPOINTMENT DURING THE PREVIOUS 3 MONTHS

PATIENT INFORMATION

2.4 Patient ID number	2.5 Sex (M/F)	2.6 Date of Birth (dd/mm/yyyy)	2.8 Drug(s) dispensed	2.9 Dose/ Frequency	2.10 Quantity	2.11 Next visit (dd/mm/yyyy)
	M F					
	M F					
	M F					
	M F					

NOTE: This data will be collected electronically where it is available.

ART stock-out form

VERIFICATION AND FACILITY INFORMATION

Data is complete and accurate	**Name**	**Signature**	**Date** (dd/mm/yyyy)
**Enumerator**			__/__/2014
**Supervisor**			__/__/2014
**Name of facility**			
**Name of storeroom contact**			
**Designation of storeroom contact**			
**Storeroom contact phone number**			

Please list dates of all stock-outs of first-round ARVs that occurred since September 1st, 2014

**Commodity**	**Stock-out start date**	**Stock-out end date**

1 PRELIMINARY INFORMATION

1.1	Interview date		__ __ / __ __ / __ __ __ __<br>D D / M M / Y Y Y Y

NAME OF FACILITY	
Name of facility	

VERIFICATION				
1.1	Start time	__ __ : __ __<br>H H : M M	End time	__ __ : __ __<br>H H : M M
Survey is complete and accurate		Name	Signature	
1.2	Enumerator			
1.3	Supervisor			

SCREENING QUESTIONS					
1.4	*After reading the consent form.*<br>Has the participant clearly read the consent form and provided informed consent?	No		1	**END**
		Yes		2	
1.5	*After reading the consent form.*<br>Has the participant agreed to have the conversation recorded?	No		1	**DO NOT RECORD**
		Yes		2	

1.6	Language of interview	English		1
		Other, specify:		98

2. PARTICIPANT INFORMATION

DEMOGRAPHICS						
2.1	Age					
2.2	Sex	Male			1	
		Female			2	
2.3	How long have you worked at this facility/office?	Years		Months		

3. BASELINE ASSESSMENT QUESTIONS FOR HEALTH FACILITY PARTICIPANTS

3.1	What is your cadre?	Doctor or Medical Licentiate		1	
		Clinical Officer		2	
		Nurse		3	
		Pharmacy staff		4	
		Other, specify: ________________		5	
3.2	At this facility, who decides the number of refill months a patient gets?				
3.3	Does this facility have a policy about the number of ARV refill months patients should get?				
3.4	***IF YES,*** *Describe policy.*				
3.5	***IF NO,*** *What most influences the decision around how many refill months to give a patient? (rank top 3 reasons)*	1.<br>2.<br>3.			
3.6	What do you think works well within this ART clinic?				
3.7	What are the main challenges you face in working in this clinic?				

3.8	Do you think it would be a good thing to provide stable patients with 3-month prescriptions? Why or why not?	
3.9	What do you think are the main challenges or issues with providing patients with 3 month refills?	
3.10	What kinds of changes would you suggest to improve the way this facility operates or how it provides care? *Prompt: Once finished initial response, ask specifically about congestion and 3 month refills.*	

4. ENDLINE ASSESSMENT QUESTIONS FOR HEALTH FACILITY PARTICIPANTS

4.1	What is your cadre?	Doctor or Medical Licentiate		1	
		Clinical Officer		2	
		Nurse		3	
		Pharmacy staff		4	
		Other, specify: ________________		5	
4.2	Are you aware that a program is being implemented at this facility to increase the proportion of stable patients on 3-month ART refills?				
4.3	Please describe any changes that have taken place in the last 4 months in the way this facility provides services to ART patients?				
4.4	What are the main challenges that you have experienced as these changes have been implemented? *Your feedback is important to improving care at this facility, so please feel free to answer honestly.*				
4.5	Have the recent changes had a positive impact on your experience and/or on the care that patients get?				

5. QUESTIONS DISTRICT PHARMACISTS

5.1	What is your position and department?	
5.2	What do you see as the main challenges in providing health facilities with the ART supplies that they request in a timely manner?	
5.3	What parts or aspects of the ART supply chain that work well?	
5.4	What do you think is a way to improve the supply system for ART drugs?	
5.6	What portion of stable ART patients in Lusaka do you think are getting 3-month prescriptions now?	
5.6	Do you think it is a good thing to provide most stable patients with multi-month prescriptions? Why or why not?	
5.8	What do you think are the main challenges or issues with providing patients with 3-month refills?	
5.9	What kinds of changes do you think would be needed to provide more patients with 3 month refills?	

6. QUESTIONS FOR MEDICAL STORES LIMITED STAFF

6.1	What do you see as the main challenges in providing health facilities with the ART supplies that they request in a timely manner?	

6.2	What parts or aspects of the ART supply chain that work well?	
6.3	What do you think is a way to improve the supply system for ART drugs?	
6.4	What portion of stable ART patients in Lusaka do you think are getting 3-month prescriptions now?	
6.5	Do you think it is a good thing to provide most stable patients with multi-month prescriptions? Why or why not?	
6.6	What do you think are the main challenges or issues with providing patients with 3-month refills?	
6.7	What kinds of changes do you think would be needed to provide more patients with 3 month refills?	

Patient Exit Interview

1 PRELIMINARY INFORMATION

1.1	Interview date		D	D	/	M	M	/	Y	Y	Y	Y

HEALTH FACILITY	
Name of facility	

VERIFICATION													
1.1	Start time	H	H	:	M	M	End time	H	H	:	M	M
Survey is complete and accurate		Name					Signature					
1.2	Enumerator											
1.3	Supervisor											

SCREENING QUESTIONS					
1.4	Is the patient under 18 years old?	No		1	**END**
		Yes		2	
1.5	Have you confirmed with the Pharmacist that the patient is on first line treatment?	No		1	**END**
		Yes		2	
1.6	*After reading the consent form.*<br>Has the patient clearly read the consent form and provided informed consent?	No		1	**END**
		Yes		2	
1.7	*After reading the consent form.*<br>Has the participant agreed to have the conversation recorded?	No		1	**DO NOT RECORD**
		Yes		2	

2 PATIENT INFORMATION

DEMOGRAPHICS OF PATIENT					
2.1	Age				
2.2	How long does it take you to get from your home to the facility?				
2.3	Unit of time	Minutes		1	
		Hours		2	
2.4	What mode of transportation do you use to get to the facility?	Walking		1	
		Biking		2	
		Minibus		3	
		Personal Car		4	
		Other, specify: _________________		98	
2.5	Sex	Male		1	
		Female		2	
2.6	What is your marital status?	Single, never married		0	
		Married, monogamous		1	
		Married, polygamous		2	
		Not married, living with partner		3	
		Widowed		4	
		Divorced/separated		5	
		Other, specify:_________________		98	
		Don't know		99	
2.7	What is the highest level of education you have completed?	No formal education		0	
		1		1	
		2		2	
		3		3	
		4		4	
		5		5	
		6		6	
		7		7	
		8		8	
		9		9	
		10		10	
		11		11	
		12		12	
		Certificate program		14	
		Other, specify:__________________		98	
		Don't know		99	

3. ART ADHERENCE AND BARRIERS TO CARE						
3.1	When were you first diagnosed with HIV?<br>*Prompt: Year of diagnosis*		Year			
3.2	When did you first start receiving care at this ART clinic? (*Month and year)*	Month	Year			
3.3	In the past month, have you missed any doses of ARV pills?	No			0	SKIP TO 3.5
		Yes			1	
		Don't know			99	SKIP TO 3.5
3.4	Why did you miss those doses?<br>**Choose all that apply, ranking those in order of importance (Do not prompt or mention options – allow the respondent to answer directly)**	Side effects			1	
		Shared pills with others			2	
		Forgot			3	
		Felt better			4	
		Too ill			5	
		Stigma, disclosure or privacy issues			6	
		Drug stockout			7	
		Lost or ran out of pills			8	
		Delivery or travel problems			9	
		Alcohol			11	
		Depression			12	
		Pill burden			13	
		Lack of food			14	
		Other (specify): ______________________			98	
		Don't know			99	
3.5	Thinking back on the previous 3 months, have you missed any scheduled appointments?	No			0	SKIP TO 3.8
		Yes			1	
		Don't know			99	SKIP TO 3. 8
3.6	If so, how many appointments did you miss?<br>**Input number, or 99 for don't know**					
3.7	If so, why?<br>**Choose all that apply (do not prompt or mention choices – allow the respondent to answer directly)**	Forgot			1	
		Felt better			2	
		Stigma/disclosure			3	
		Too ill			4	
		Lack of money			5	

		Couldn't get out of school or work		6	
		Disliked patient experience at the facility		7	
		Facility was too far		8	
		Didn't have money for transport		9	
		Went to a different facility		10	
		Other (specify):_______________________		98	
		Don't know		99	
3.8	What are the main difficulties that you face in receiving care at this clinic?<br><br>Choose all that apply.	Transportation/distance to facility		1	
		Taking time off from school/work to go to facility		2	
		Waiting times at facility		3	
		Stigma/disclosure issues		4	
		Side effects of medication		7	
		Short refill period		8	
		Other (specify): ___________		98	
		Don't know		99	
4. ART CARE					
4.1	On a scale of 1 to 5, 1 being very unhappy and 5 being very happy, how happy are you with how the health workers at the facility interacted with you today?	Very unhappy		1	
		Somewhat unhappy		2	
		Neutral		3	
		Somewhat happy		4	
		Very happy		5	
		Don't know		99	
4.2	What are the main reasons you choose to come to this facility, as opposed to another facility, for care?<br><br>Choose all that apply	This facility is closest to me		1	
		This facility has less wait time than other facilities		2	
		This facility is stocked/usually stocked with drugs		3	
		I like the healthcare workers at this facility		4	
		I have friends who come to the facility		5	
		I like the counseling sessions at the facility		6	
		I like the fact that families come on the same day		7	
		Other (specify): ______________________		98	

4.3	What are the factors that you think work well in this clinic?	List most common			
4.4	Do you have any concerns about getting 3 months of ART drugs at the same time?<br>**Choose all that apply (Do not prompt or mention options – allow the respondent to answer directly)**	Losing medication		1	
		Forgetting to come back after 3 months		2	
		Facility will run out of drugs if everyone gets 3 months		3	
		Not having a safe place to store drugs		4	
		Not being able to see a doctor or nurse for 3 months		5	
		Other (specify): ______________________		98	
		Don't know		99	
4.5	Do you prefer to receive 3 or more months of ART drugs at a time?	Yes, I prefer to receive three or more months of drugs at a time		0	
		No, I prefer to receive a smaller amount of drugs at a time		1	
		Don't know		99	
4.6	Are there any reasons why it is good to receive 3 months of ART drugs at a time?<br>**Choose all that apply (Do not prompt or mention options – allow the respondent to answer directly)**	Not having to come back to clinic as often		1	
		Knowing that I have the medication I need for at least 3 months		2	
		Not having to wait in line as long at the pharmacy because there are not as many patients coming each day		3	
		Other (specify): ______________________		98	
		Don't know		99	

QUESTIONS TO BE USED IN ENDLINE, POST-INTERVENTION SURVEY ONLY, ONLY FOR THOSE PATIENTS IN FACILITIES THAT RECEIVED AN INTERVENTION (IN ADDITION TO ABOVE)

4.7	Have there been any changes in the care that you have received at this facility in the last 4 months?	No		0	SKIP TO 4.6
		Yes		1	
		Don't know		99	SKIP TO 4.6
4.8	Please describe any changes that have happened in the care that you receive?				
4.9	Did you receive ART drugs during this	No		0	SKIP

	visit?				TO 4.8
		Yes		1	
		Don’t know		99	SKIP TO 4.8
4.10	How many months of ART drugs did you receive?	Less than one month		0	SKIP TO 4.10
		One month		1	
		Two months		2	
		Three months		3	
		More than three months		4	
		Don’t know		99	SKIP TO 4.10
4.11	Have you ever received 3 or more months of ART drugs during one visit in the past?	No		0	
		Yes		1	
		Don’t know		99	
4.12	During your visit today, did a doctor, nurse or pharmacist talk to you about the importance of taking your ARTs on schedule?	No		0	
		Yes		1	
		Don’t know		99	
4.13	Are you aware that your clinic is making an effort to improve the efficiency of the clinic and give you 3 month rather than shorter refills?	No		0	
		Yes		1	
4.14	How does receiving a 3 month rather than a shorter refill change your experience in receiving care at this facility?				

CONSENT FORM

This form means you can say NO

My name is ____ and …
Print your name

- I have gone through the Decongestion flipchart / participant information sheet
- I understand that I will be answering questions about successes and failures at the ART Clinic and that the interview will take approximately 10 minutes to complete
- I know its OK to say NO
  (I can say NO to all or any part of the interview and even if I do say YES, I can change my mind later and say NO)
- I know all information collected will be kept strictly confidential
- I know there are no direct benefits for participation and I will not be paid
- The benefits of study involvement are for the wider community if we can improve the way Zambia delivers ART care in the future

I give my permission to give an interview: *(please tick Yes or No)*

Yes	No
☐	☐

I give my permission to have the interview recorded: *(please tick Yes or No)*

Yes	No
☐	☐

………………………………	………………………………	………………………………
Participant's signature	Team Member's signature	Interpreter signature
______ / ______ / ______ Date	______________________ Print Team Member Name	______________________ Print Interpreter Name *(Write N/R if not required)*

………………………………………………………………………………………………

(tear away this section for participant to take away if they want it)

Issues or concerns

Please feel free to contact our study team if you have any questions about the study.

Felton Mpasela (+260 977 521 124)

If you have any concerns or complaints about your rights or the conduct of the study you may contact the Secretary of the ERES Ethics Committee at 0955155633.

CONSENT FORM

This form means you can say NO

My name is ____ and …
Print your name

- I have gone through the decongestion flipchart / participant information sheet
- I understand that I will be answering questions about my time at the ART Clinic and that the interview will take approximately 10 minutes to complete
- I know its OK to say NO
  (I can say NO to all or any part of the interview and even if I do say YES, I can change my mind later and say NO)
- I know all information collected will be kept strictly confidential
- I know that I will be asked questions about the difficulties in staying in ART care and that might make me feel uncomfortable
- I know there are no direct benefits for participation and I will not be paid
- The benefits of study involvement are for the wider community if we can improve the way Zambia delivers ART care in the future

I give my permission to give an interview: *(please tick Yes or No)*

Yes	No
☐	☐

I give my permission to have the interview recorded: *(please tick Yes or No)*

Yes	No
☐	☐

....	....	....
Participant's signature	Team Member's signature	Interpreter signature
______ / ______ / ______ Date	______________ Print Team Member Name	______________ Print Interpreter Name *(Write N/R if not required)*

....

(tear away this section for participant to take away)

Issues or concerns

Please feel free to contact our study team if you have any questions about the study.

Felton Mpasela (+260 977 521 124)

If you have any concerns or complaints about your rights or the conduct of the study you may contact the Secretary of the ERES Ethics Committee at 0955155633.

INFORMATION SHEET FOR RESEARCH PARTICIPANTS

Study Title: Determining the effectiveness of improved clinic efficiency and supply chain quality on the proportion of stable patients on 3-month refills at 4 months post intervention: A pair-matched, randomised, difference in difference study

Invitation Paragraph

In Zambia, rapid ARV refills (due to refill script <3 months) and long wait times can lead to congested ART facilities, decreased ART retention, and poor treatment outcomes. The reason for the provision of shorter refills appears to be multifactorial. Some pharmacists are concerned about stock outs and ration ARTs by providing shorter refills, while others make ad hoc decisions in lieu of a standard operating procedure that specifies a number of refill months. This study will investigate whether clinic efficiency and supply chain quality improvement increases the number of ART patients with 3-month refills with the hope of decreasing the ART congestion. We invite you to be interviewed as part of this study. Before we begin, please read this information sheet and decide whether or not you want to participate.

What is the purpose of the study?

The primary purpose of this study is to determine whether clinic efficiency and supply chain quality improvement increases the number of stable ART patients who receive 3-month refills. Additionally, we will investigate whether clinic efficiency and supply chain quality improvement also affects patient wait times, ART retention, and the ratio of staff to patients.

Why have I been invited to take part?

You are invited to participate because your experiences as a health care worker in this facility are valuable and we believe you can provide important information to us that may be relevant to our research.

Do I have to take part?

Your participation is voluntary. We believe that your contribution to this study would be valuable, but if you do not wish to participate you are allowed to say no.

What will happen to me if I take part?

If you agree to participate in the study, we will ask you to read the rest of this information sheet and sign a consent form. When this is completed, a member from our study team will conduct a 10 minute interview and ask you basic questions about your experience and perception of this facility, such as your thoughts on the patient experience and process successes and failures. You are allowed to withhold an answer to any question you are not comfortable answering.

What are the possible risks of taking part?

There is little to no risk associated with taking part in this study. All the information that you provide to us during the interview will be stored without your name and be kept in a confidential location. Therefore, no one will be able to link you to your answers. Only members of this study team will have access to the information that you provide to us during your interview. We would like to record our conversation, but will only do so if you agree.

What are the possible benefits of taking part?

While there are no immediate and personal benefits to your participation in this study, your involvement could eventually contribute to public health care system improvement in Zambia, as well as improved ART patient outcomes.

<u>Will my taking part be kept confidential?</u>

All information that you provide to us will be kept confidential. Your name will never be recorded with the information that you provide to us, only the name of the health facility where you work. Only members of this study team will have access to the information that you provide to us. Information emanating from this study will only be made public in a completely unattributable format to ensure that no participant can be identified.

<u>How is the project being funded?</u>

The study is funded by the British Department for International Development (DFID) and managed by the Ministry of Health. In Zambia, Clinton Health Access Initiative plays a supporting role to the Ministry of Health.

<u>What will happen to the results of the study?</u>

The results of this study will be analysed and made public by the Ministry of Health. If evidence from this study shows that the intervention provides a significant benefit to patients, national guidelines could be revised by the Ministry of Health in Zambia.

<u>What if I have further questions, or if something goes wrong?</u>

If you have any questions or if this study has harmed you in any way and you wish to make a complaint about the conduct of the study, you can contact the team using the following contact details:

Elizabeth McCarthy
emccarthy@clintonHealthAccess.org
+260 979 381 709

Thank you for reading this information sheet and for considering taking part in this research.

The ARV Refill Study

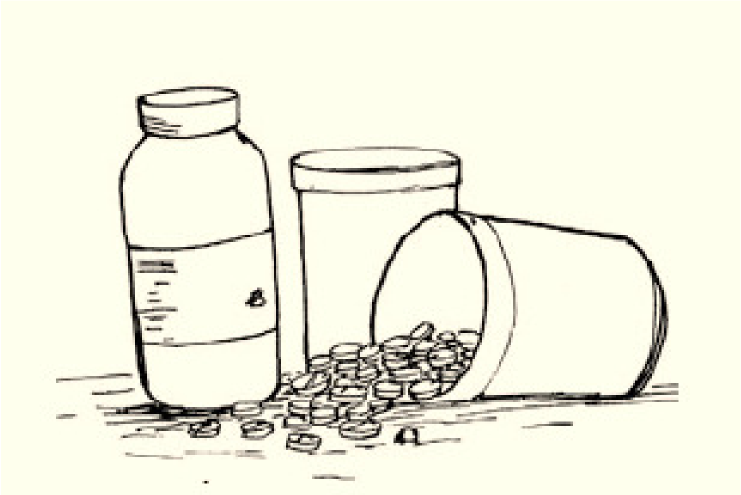

This study aims to measure how health facility efficiency and supply chain quality improvement changes your ARV refill length. Talking to us does not mean that you have to be involved in this study.

What is the study?

This project is looking at people who are receiving ART. We want to find out if recent changes at your health facility have had an impact on the amount of time between your ARV refills. Additionally, we want to see if there have been changes to your wait time and overall clinic experience.

IF YOU SAY YES

- I will conduct a 10 minute interview with you
- I will be asking questions that relate to your HIV status and experience with getting your ARVs, and this might make you uncomfortable
- There is no payment or direct benefit for you, but your involvement could improve services at ART clinics for all Zambians
- At the end of the project, we will present the results to the Ministry of Health and they will decide if they want to make changes at all public facilities in Zambia

IF YOU SAY NO

- This is a study and you are allowed to say no if you don't want to participate and there will be no consequences to saying no.

How do we store information and keep it safe?

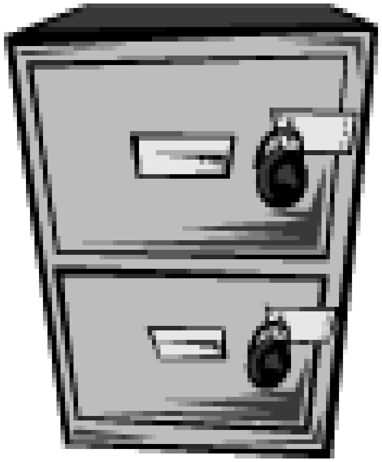

- All records from the project are strictly confidential and will be stored in a locked cupboard in a locked office
- Only authorised people will have access to the records in the locked cupboard
- The records will not have your name written and no one will be able to tell it was you who gave answers to the questions during the interview

If you want to take part in the project, you need to:

- Sign the form to give consent (you must be older than 18 years old to participate)
- Take 10 minutes and participate in an interview where I ask you questions and write down your answers to my questions and I will record the conversation only if you are happy for that.
- Remember, you can change your mind at any time by asking me to stop, and you can decide not answer some questions as well
- Do you have any questions before we start?

Mwango Albert, Dr. BSc HB, MBChB, MPH **April 18th 2013**

CURRICULUM VITAE

DATE OF BIRTH: 25th March 1974
NATIONALITY: Zambian
NATIONAL REGISTRATION No.: 318530/67/1
PASSPORT No.: ZP000189
E-MAIL: mwangoaj@yahoo.co.uk; albert.mwango@moh.gov.zm
Cell (Zambia): +260-950-230-522

QUALIFICATIONS

MASTERS OF PUBLIC HEALTH (MPH), VANDERBILT UNIVERSITY SCHOOL OF MEDICINE, NASHVILLE TN, USA

Period: September 2010 to May 11 2012.

BACHELOR OF MEDICINE AND BACHELOR OF SURGERY (MBChB), SCHOOL OF MEDICINE UNIVERSITY OF ZAMBIA

BACHELOR OF HUMAN BIOLOGY (BSc HB), SCHOOL OF MEDICINE UNIVERSITY OF ZAMBIA

Period: February 1995 to January 2002.

EDUCATION

IGCSE 'O' Level Certificate, 1992, Simba International School Ndola, Zambia

Subjects: Mathematics, French, English, Business Studies, Biology, Physics, Chemistry, and Computer Studies.

CAREER PLAN

I am employed as the National Antiretroviral Programme Coordinator and HIV/STI specialist for Zambia at the Ministry of Health. I intend to follow this up with a PhD in Global Health with a focus on Health Informatics and HIV medicine.

WORK EXPERIENCE

CURRENT JOB EXPERIENCE

Ministry of Health (MOH) and Central Board of Heath (CBOH), Zambia
Title: *National Antiretroviral Programme Coordinator*

Responsible to: Director Clinical Care & Diagnostic Services
Duties: Planning and coordinating the implementation of the ARV programme in the country; assisting in formulating and interpreting policy guidelines, developing strategies for strengthening systems in the administration of antiretroviral drugs; monitoring and evaluating antiretroviral therapy programme implementation; facilitating training of health personnel.

Period: May 24th 2004 – 23rd May 2006 (CBOH); 24th May 2006 to date (MOH)

PREVIOUS JOB EXPERIENCE

1. Ndola Central Hospital
Title: *Senior Resident Medical Officer and ART Centre Coordinator*

Responsible to: Director Clinical Services
Duties: Manning Emergency (Casualty) Room and OPD activities; Antiretroviral Centre Committee member and; Antiretroviral Therapy clinic coordinator and medical officer in the introductory public sector pilot programme for Antiretroviral Therapy at Ndola Central Hospital. During which 850 patients were commenced and monitored on ART.

Number of Subordinates: ten (10) Clinical officers
Period: April 28, 2003 – May 23, 2004

2. Ndola Central Hospital
Title: *Junior Resident Medical Officer*

Responsible to: Director Clinical Services/Heads of Department
Duties: Rotations in the Departments of Medicine (3 months), Paediatrics (3 months), Obstetrics and Gynaecology (7 months)

Number of Subordinates: six (06)
Period: February 2002- to April 28, 2003

WORKSHOPS AND COURSES

Regional consultative meetings on development of strategies for implementing antiretroviral therapy programmes in resource poor settings; strategic meetings for developing proposals for Global Fund, World Bank (ZANARA), USG PEPFAR, Italian Fund/WHO, UNITAID/UNICEF, UNITAID/Clinton Foundation; trainings for clinicians, pharmacovigilance, programme monitoring and evaluation and nutrition interventions in HIV/AIDS. Providing expert consultative input to WHO Headquarters strategies; UAB Summer Institute of Public Health; UAB Scientific Papers Writing course;

CONSULTANT SERVICES

1. "Zambia Contextualization of HIV and AIDS Treatment Literacy Toolkit for Communities"; Employer Name: Southern Africa HIV/AIDS Information Dissemination Service, April 1, 2008 – May 10, 2008
2. "Review and provision of expert advice on content for print, radio and television materials on HIV and AIDS in Children and Alcohol Abuse"; Employer Name: Zambia Centre for Communication Programmes; August 16th – December 2008
3. "Adaptation of standard operating Procedures for Antiretroviral therapy and Adherence counselling for use in public institutions in Zambia"; Employer Name: Family Health International; September 16-27th 2007
4. "Review of Antiretroviral therapy Available in Zambia 2007"; Employer Name: Refugee Legal Centre, United Kingdom; September 17-18th 2007
5. "Country Assessment of Zambia Diflucan Partnership Programme"; Employer Name: Johns Hopkins Programme for Information and education for Gynaecology and Obstetrics Corporation; September 14 2006– December 31st, 2006
6. "Zambia Contextualization of HIV and AIDS Treatment Literacy Toolkit for Women"; Employer Name: Southern Africa HIV/AIDS Information Dissemination Service, November 20, 2006 – November 30, 2006
7. "Evaluation of the Zambia National Antiretroviral Therapy Implementation process (2004-2005) and development of a three year implementation plan (2006-2008)"; Employer Name: Abt Associates; January 30, 2006 - April 30, 2006.

8. “Policy and Practice Harmonisation review of Kwatu Radio Drama” ; Employer Name: Zambia Centre for Communication Programmes; June 23, 2005 – July 8, 2005.

ONGOING STUDIES OR PROJECTS

1. Lamivudine (3TC) and Emtricitabine (FTC) in ARV Treatment Regimens in Resource-Limited Settings observational study; Sponsors Ministry of Health Zambia, United States Centers for Disease Control and Prevention (CDC)
2. Scaling Up TB Prevention, Screening, diagnosis and Care in Zambia, Implementing the WHO 3 I’s in Zambia; Sponsors United States Centers for Disease Control and Prevention (CDC)
3. Evaluation of an Integrated Community-Based and Clinical HIV/AIDS Program in Sinazongwe District, Zambia; Sponsor: United States Centers for Disease Control and Prevention (CDC)

ABSTRACTS (Oral Presentation)*

1. Eric Fleutelot and **Albert Mwango**. *IAS TasP regional consultation report*. 3rd International HIV Workshop on Treatment as Prevention. Vancouver, BC, Canada. April 22-25, 2013. Late Breaker “ACCEPTED”.

2. Izukanji Sikazwe, **Albert Mwango**, Kenichi Komada, Shinsuke Miyano, Callie Scott, Gardner Syakantu. *A cost analysis of the expansion of antiretroviral treatment to the rural health centre level through mobile HIV services in Zambia*. 7th IAS Conference on HIV Pathogenesis, Treatment and Prevention 30 June -3 July 2013, Kuala Lumpur, Malaysia. “ACCEPTED”.

3. **Albert Mwango**, Izukanji Sikazwe, Kenichi Komada, Shinsuke Miyano, Gardner Syakantu. *Outcomes of national expansion program for antiretroviral treatment to rural health centre level through mobile HIV services in Zambia*. 7th IAS Conference on HIV Pathogenesis, Treatment and Prevention 30 June -3 July 2013, Kuala Lumpur, Malaysia. “ACCEPTED”.

4. Lloyd Mulenga, **Albert Mwango**, Patrick Musonda, Mary-Ann Davies, Aggrey Mweemba, Alexandra Calmy, Jeffrey Stringer, Olivia Keiser, Benjamin Chi, Gilles Wandeler. Renal Function and Outcomes of Tenofovir-Containing ART in Zambia. *20thConference on Retroviruses and Opportunistic Infections, Atlanta, USA*. March 3-6, 2013; Paper 816 (M-142).

5. Michelle Li, Brad Guffey, Patrick Musonda, Izukanji Sikazwe, **Albert Mwango**, Benjamin Chi, Jeffrey Stringer. ART initiation substantially improves retention among patients enrolling in care in Lusaka, Zambia. *20thConference on Retroviruses and Opportunistic Infections, Atlanta, USA*. March 3-6, 2013; Paper 93.

6. Albert Mwango, Benjamin H Chi, Douglas C Heimburger, Sten H Vermund. Program retention efficiency in the antiretroviral therapy program in Zambia: A retrospective cohort study. *Sothern African HIV clinicians Society Conference 2012.*

7. C. Bositis, D. Patel, S. Lakhi, **A. Mwango**, K. Bowa, M. Hossain, S. Schneider, R. Sheneberger. Scaling up local human resource capacity: experience of the Zambian HIV residency program. : IAS 2010 Abstract no. WEPE0862 "

8. B. Chi, A. Westfall, M. Fox, S. Phiri, H. Prozesky, L. Fairall, **A. Mwango**, A. Boulle, M. Egger, J. Stringer, M. Brinkhof, O. Keiser. Empirically defining lost to follow-up for antiretroviral therapy programs in Southern Africa. : IAS 2010: Abstract no. THPE0422 "

9. * **Mwango A**, Giganti M, Mulenga L, Reid S, Chisembele-Taylor A, Chintu N, Chi B, Stringer E, Stringer J. First-line tenofovir ART in Zambia. *16thConference on Retroviruses and Opportunistic Infections, Montreal, Canada*. February 8-11, 2009; Abstract 142.

10. Stringer J, Mulenga L, Giganti M, Reid S, Chisembele-Taylor A, Chintu N, Chi B, Stringer E, **Mwango A**. Effectiveness of generic vs. proprietary first-line ARV regimens in a primary health care setting: Lusaka, Zambia. *16th Conference on Retroviruses and Opportunistic Infections, Montreal, Canada*. February 8-11, 2009; Abstract 611.

11. L. Luwatula, R. Hughes, E. Sinyinza, **A. Mwango**. Strengthening pre-service education in Zambia for the provision of HIV/AIDS services and care. : *AIDS 2008 - XVII International AIDS Conference*: Abstract no. MOPE0848"

12. Sinkala M, Levy J, Zulu I, **Mwango A**, Stringer E, Chi B, Reid S, Ellerbrock T, Bulterys M, Stringer J. Rapid scale-up of antiretroviral services in Zambia: 1-year clinical and immunological outcomes. *13th Conference on Retroviruses and Opportunistic Infections in Denver, CO*,USA. February 5-8, 2006; Abstract 64.

13. L. Luwatula, R. Hughes, E. Sinyinza, **A. Mwango**. Addressing human resources: results of a crash program to train students in Zambia before graduation. : *AIDS 2006 - XVI International AIDS Conference*: Abstract no. CDD1280

PUBLICATIONS (Co-Author)

1. Marseille E, Giganti MJ, **Mwango A**, Chisembele-Taylor A, Mulenga L, Over M, Kahn JG, Stringer JS. *Taking ART to scale: determinants of the cost and cost-effectiveness of antiretroviral therapy in 45 clinical sites in Zambia*. Source Health Strategies International, Oakland, CA, USA. emarseille@comcast.net

2. Giganti MJ, Limbada M, **Mwango A**, Moyo C, Mulenga LB, Guffey MB, Mulenga PL, Bolton-Moore C, Stringer JS, Chi BH. *Six-month hemoglobin concentration and its association with subsequent mortality among adults on antiretroviral therapy in Lusaka, Zambia*. J Acquir Immune Defic Syndr. 2012 Sep 1;61(1):120-3. PubMed PMID: 22659648.

3. Koethe JR, Blevins M, Bosire C, Nyirenda C, Kabagambe EK, **Mwango A**, Kasongo W, Zulu I, Shepherd BE, Heimburger DC. *Self-reported dietary intake and appetite predict early treatment outcome among low-BMI adults initiating HIV treatment in sub-Saharan Africa.* Public Health Nutr. 2012 Jun 13:1-10. [Epub ahead of print] PubMed PMID: 22691872.

4. Stringer JS, **Mwango A**, Giganti MJ, Mulenga L, Levy JW, Stringer EM, Mulenga P, Saag MS, Musonda P, Williams FB, Reid SE, Chi BH. *Effectiveness of generic and proprietary first-line anti-retroviral regimens in a primary health care setting in Lusaka, Zambia: a cohort study*. Int J Epidemiol. 2012 Apr;41(2):448-59. PubMed PMID: 22493326; PubMed Central PMCID: PMC3324461.

5. Chi BH, **Mwango A**, Giganti MJ, Sikazwe I, Moyo C, Schuttner L, Mulenga LB, Bolton-Moore C, Chintu NT, Sheneberger R, Stringer EM, Stringer JS. *Comparative outcomes of tenofovir-based and zidovudine-based antiretroviral therapy regimens in Lusaka, Zambia.* J Acquir Immune Defic Syndr. 2011 Dec 15;58(5):475-81. PubMed PMID: 21857354; PubMed Central PMCID: PMC3215810.

6. Chi BH, Yiannoutsos CT, Westfall AO, Newman JE, Zhou J, Cesar C, Brinkhof MW, **Mwango A**, Balestre E, Carriquiry G, Sirisanthana T, Mukumbi H, Martin JN, Grimsrud A, Bacon M, Thiebaut R; International Epidemiologic Databases to Evaluate AIDS Collaboration. *Universal definition of loss to follow-up in HIV*

treatment programs: a statistical analysis of 111 facilities in Africa, Asia, and Latin America. PLoS Med. 2011 Oct;8(10):e1001111. Epub 2011 Oct 25. PubMed PMID: 22039357; PubMed Central PMCID: PMC3201937.

7. Koethe JR, Blevins M, Nyirenda C, Kabagambe EK, Shepherd BE, Wester CW, Zulu I, Chiasera JM, Mulenga LB, **Mwango A**, Heimburger DC. *Nutrition and inflammation serum biomarkers are associated with 12-week mortality among malnourished adults initiating antiretroviral therapy in Zambia.* J Int AIDS Soc. 2011 Apr 10;14:19. PubMed PMID: 21477359; PubMed Central PMCID: PMC3094357.

8. Schöni-Affolter F, Keiser O, **Mwango A**, Stringer J, Ledergerber B, Mulenga L, Bucher HC, Westfall AO, Calmy A, Boulle A, Chintu N, Egger M, Chi BH; Swiss HIV Cohort Study; IeDEA Southern Africa. *Estimating loss to follow-up in HIV-infected patients on antiretroviral therapy: the effect of the competing risk of death in Zambia and Switzerland.* PLoS One. 2011;6(12):e27919. Epub 2011 Dec 19. PubMed PMID: 22205933; PubMed Central PMCID: PMC3242760.

9. Chi BH, **Mwango A**, Giganti M, Mulenga LB, Tambatamba-Chapula B, Reid SE, Bolton-Moore C, Chintu N, Mulenga PL, Stringer EM, Sheneberger R, Mwaba P, Stringer JS. Early clinical and programmatic outcomes with tenofovir-based antiretroviral therapy in Zambia. J Acquir Immune Defic Syndr. 2010 May 1;54(1):63-70. PubMed PMID: 20009765; PubMed Central PMCID: PMC2862003.

10. Chi BH, Cantrell RA, **Mwango A**, Westfall AO, Mutale W, Limbada M, Mulenga LB, Vermund SH, Stringer JS. *An empirical approach to defining loss to follow-up among patients enrolled in antiretroviral treatment programs.* Am J Epidemiol. 2010 Apr 15;171(8):924-31. Epub 2010 Mar 10. PubMed PMID: 20219765; PubMed Central PMCID: PMC2850972.

11. Chi BH, Cantrell RA, Zulu I, Mulenga LB, Levy JW, Tambatamba BC, Reid S, **Mwango A**, Mwinga A, Bulterys M, Saag MS, Stringer JS. *Adherence to first-line antiretroviral therapy affects non-virologic outcomes among patients on treatment for more than 12 months in Lusaka, Zambia.* Int J Epidemiol. 2009 Jun;38(3):746-56. Epub 2009 Feb 17. PubMed PMID: 19223334; PubMed Central PMCID: PMC2689395.

12. Morris MB, Chapula BT, Chi BH, **Mwango A**, Chi HF, Mwanza J, Manda H, Bolton C, Pankratz DS, Stringer JS, Reid SE. *Use of task-shifting to rapidly scale-up HIV treatment services: experiences from Lusaka, Zambia.* BMC Health Serv Res. 2009 Jan 9;9:5. PubMed PMID: 19134202; PubMed Central PMCID: PMC2628658.
13. Torpey KE, Kabaso ME, Mutale LN, Kamanga MK, **Mwango A**, Simpungwe J, Suzuki C, Mukadi YD. *Adherence support workers: a way to address human resource constraints in antiretroviral treatment programs in the public health setting in Zambia.* PLoS One. 2008 May 21;3(5):e2204. PubMed PMID: 18493615; PubMed Central PMCID: PMC2377331.

14. Bolton-Moore C, Mubiana-Mbewe M, Cantrell RA, Chintu N, Stringer EM, Chi BH, Sinkala M, Kankasa C, Wilson CM, Wilfert CM, **Mwango A**, Levy J, Abrams EJ, Bulterys M, Stringer JS. *Clinical outcomes and CD4 cell response in children receiving antiretroviral therapy at primary health care facilities in Zambia.* JAMA. 2007 Oct 24;298(16):1888-99. PubMed PMID: 17954540.

15. Huff-Rousselle M, Simooya O, Kabwe V, Hollander I, Handema R, **Mwango A**, Mwape E. *Pharmacovigilance and new essential drugs in Africa: Zambia draws lessons from its own experiences and beyond. Glob Public Health.* 2007;2(2):184-203. PubMed PMID: 19280399.

16. Stringer JS, Zulu I, Levy J, Stringer EM, **Mwango A**, Chi BH, Mtonga V, Reid S, Cantrell RA, Bulterys M, Saag MS, Marlink RG, Mwinga A, Ellerbrock TV, Sinkala M. *Rapid scale-up of antiretroviral therapy at primary care sites in Zambia: feasibility and early outcomes.* JAMA. 2006 Aug 16;296(7):782-93. PubMed PMID: 16905784.

SARAH MOBERLEY

Contact Details: Personal email: sarah.moberley@gmail.com
Telephone: +256 (0) 787690512

Education

3/2005 – 08/2009	PhD (Doctor of Philosophy in Public Health), University of Melbourne, Australia
Ph.D Title	"Use and Effectiveness of the 23-valent Pneumococcal Polysaccharide Vaccine in Indigenous Adults"
2004	Immunisation for Registered Nurses, New South Wales College of Nursing
2/1999 - 11/2002	Masters of Public Health, James Cook University, Australia
2/1994 – 11/1996	Bachelor of Nursing, University of Sydney, Australia<br>Registered with Australian Health Practitioner Regulation Agency (NMW0001820709)

Employment History

12/2013 to present Senior Technical Advisor, Applied Analytics Team, Clinton Health Access Initiative, Kampala, Uganda

Full time position responsible for the management of a US$3million project to support the Ugandan and Zambian Ministries of Health in accelerating evidence based decision making. The project conducts rapid and rigorous evaluations which are driven by the Ministry of Health; the findings are then catalysed into health policy where appropriate.

03/2013 to 12/2013 Consultant Epidemiologist, Vaxtracker Project, Newcastle, Australia

Consultant Epidemiologist for 'Vaxtracker'; a pilot program to test a web based survey of adverse events following vaccination in children. Position responsible for; data analysis (Stata), classification of adverse events, preparation of reports, collation of background data to determine evidence for signal detection and recommendations for future use of the program.

11/2010 to 12/2013 Post-Doctoral Fellow, Menzies School of Health Research, Darwin, Northern Territory, Australia

Part time position responsible for conducting research in the area of vaccine preventable infections, particularly pneumococcal disease and pneumococcal vaccines.

Key Achievements:

- Coordinating Author of the Cochrane Review of pneumococcal vaccines in adults
- Management of study into 23-valent pneumococcal polysaccharide vaccine in Indigenous adults
- Conducted analysis and writing up of "PneuMum": A randomised controlled trial of maternal pneumococcal vaccination for protection of Indigenous children from ear disease
- Publication and presentation of research findings (see section below)
- Supervision of data analysis for Masters student

08/2010 to 08/2012 Senior Policy Analyst, Immunisation Unit, New South Wales State Department of Health, Australia

Provision of expert advice regarding the New South Wales State Immunisation Program to ensure the safe and effective delivery of vaccines to the State.
Key Achievements:

- Managed the New South Wales State policy to improve the timeliness of immunisation coverage of Aboriginal children and prepared a culturally appropriate submission for the employment of 12 Aboriginal Health workers to undertake the project
- Investigation of two vaccine safety alerts (intussusception following rotavirus vaccine and increase in severe local reactions to pneumococcal polysaccharide vaccine)

09/2009 to 06/2010 Epidemiologist, Papua New Guinea Institute of Medical Research, Goroka, PNG

Responsible for research projects within the Department of Acute Respiratory Infections, including the supervision of Masters students and training of staff in epidemiology and research methods.
Key Achievements:

- Established whole of life pneumonia prevalence and aetiology study
- Conducted the training of Masters students in epidemiology and biostatistics
- Supervised two Masters students
- Conducted data analysis on the efficacy of neonatal pneumococcal vaccination against pneumococcal nasopharyngeal carriage

01/2009 to 06/2009 Epidemiologist, Médecins Sans Frontières, Amsterdam, Holland

Responsible for epidemiological support to field projects related to vaccine preventable diseases, nutrition, and operational research.
Key Achievements:

- Conducted mortality, nutrition and food security study for refugee population in Bangladesh
- Assisted in response to, and training of local staff in management of meningitis outbreak, northern Nigeria

03/2005 to 12/2008 Quality Control Auditor, Menzies School of Health Research, Northern Territory, Australia

Responsible for the preparation and conduct of clinical research including; trial protocol preparation, developed standard operating procedures, ethics application, data collection forms, data management and analysis, vaccine logistics, file tracking, ensure compliance with Good Clinical Practice. Conducted data analysis and communication of key findings (see section below).

09/2006 Project Coordinator, Médecins Sans Frontières, Humera, Ethiopia

Emergency response to measles and cholera outbreak within existing HIV/ Kala-azar project.
Key Achievements:

- Management of mass measles vaccination campaign in adults within Humera Woreda in collaboration with Ministry of Health
- Developed training materials and established evaluation mechanisms
- Trained supervisors and vaccination teams in best practice mass measles vaccination
- Conducted field visits to ensure technical compliance with best practice, appropriate community mobilisation and education, and high coverage in target population

08/2005 Project Coordinator, Médecins Sans Frontières, Niger

Responsible for health/ nutrition assessment, establishment of a therapeutic feeding centre for severely malnourished children and mass measles vaccination campaign.

Key Achievements:

- Rapid nutritional and health assessment
- Recruited and trained national staff for therapeutic feeding centre
- Trained first mission expat staff

07/2004 to 02/2005 Medical Team Leader, Médecins Sans Frontières, Lira, Uganda

Responsible for the delivery of primary health and nutrition services to population residing in internally displaced camps.

Key Achievements:

- Implemented country health policy, including evaluation of medical and nutritional regional programs including therapeutic and supplementary feeding, primary health care, tuberculosis and HIV programs and emergency preparedness
- Recruited and provided support and training to medical expatriate and national staff (13 medical expatriate, >300 national staff)
- Collation and analysis of routine and survey data (medical and nutritional)
- Developed, implemented and monitored medical and nutritional protocols and guidelines
- Contributed to country medical reports, annual plan, proposals and donor reporting
- Responded to disease outbreaks as identified by surveillance (containment of measles outbreak by mass vaccination)

01/2004 to 06/2004	Clinical Nurse Consultant, Illawarra Area Health Service, Australia
07/2003 to 12/2003	Public Health Officer, Wentworth Area Health Service, Nepean, Australia
04/2003 to 06/2003	Health Manager, International Rescue Committee, Iraq
02/2003 to 04/2003	Project Coordinator, International Medical Corps, Ethiopia
05/2002 to 02/2003	Public Health Officer, Wentworth Area Health Service, Nepean, Australia
11/2001 to 05/2002	Public Health Nurse, Medical Emergency Relief International, Afghanistan
05/2001 to 10/2001	Emergency Drought Relief Coordinator, Médecins Sans Frontières, Afghanistan
10/2000 to 05/2001	Immunisation Coordinator and Public Health Officer, Wentworth Area Health Service, Nepean, Australia
1999 to 2000	Microbiology Surveillance and Liaison Nurse, Westmead Children's Hospital, Sydney, Australia
1998 to 2000	Registered Nurse, Casual Pool, Royal Prince Alfred Hospital, Sydney, Australia
04/1998 to 10/1998	Paediatric Nursing Sister in Charge, St Anthony's Hospital, Tororo, Uganda
01/1997 to 12/1997	Registered Nurse, Graduate Program, St Vincent's Private Hospital, Australia

Consultancy

Indigenous Generational Reform, Stage 1: Early Child Development and Growth. A review of the literature on the effectiveness of population-based preventative interventions. Northern Territory Government. January 2007.

Publications

Moberley S, Andrews R. An evaluation of the indirect cohort method to estimate the effectiveness of the pneumococcal polysaccharide vaccine. Journal of Vaccines and Immunization, 2014, 2(1):4-6.

Moberley S, Holden J, Tatham M, Andrews R. Vaccines for preventing pneumococcal infection in adults. Cochrane Database of Systematic Reviews, Issue 1, 2013.

Moberley S, Torzillo P. Pneumococcal polysaccharide vaccine in high risk adults, BMJ, 2010; 340:1139 (editorial).

Moberley S, Krause V, Cook H, Mullholland K, Carapetis J, Torzillo P, Andrews R. Failure to vaccinate or failure of vaccine? Effectiveness of the 23-valent pneumococcal polysaccharide vaccine in Indigenous adults in the Northern Territory. Vaccine, 2010; 28(11): 2296-2301.

Moberley S, Holden J, Tatham M, Andrews R. Vaccines for preventing pneumococcal infection in adults. Cochrane Database of Systematic Reviews, Issue 1, 2008.

Quinn H, Wood N, Cannings K, Dey A, Wang H, Menzies R, *Moberley S*, Reid S, McIntyre P, Macartney K. Intussusception following monovalent human rotavirus vaccine in Australia: severity and comparison of using healthcare database records versus case-confirmation to assess risk. Paediatric Infectious Diseases Journal, Pediatr Infect Dis J. 2014 Apr 18.

Andrews R, *Moberley S*. The controversy over the efficacy of the pneumococcal vaccine. Canadian Medical Association Journal, 2009; 180(1):18-9 (commentary).

Andrews R, *Moberley S*. Efficacy of pneumococcal polysaccharide vaccine. Canadian Medical Association Journal, 2009;180(11):1135 (letter).

Menzies R, McIntyre P, Reid R, O'Brien K, Santosham M, Watt J, Angeles G, Brown A, Dunbar M, Leach A, Crengle S, Lennon D, Mason H, Grim C, Nolan L, Smith P, Dumaresq G, Richardson R, *Moberley S*, Stirling J, Gooda M, Green M. Vaccine preventable diseases in Indigenous populations—International perspectives. Satellite Symposium of the 5th International Symposium on Pneumococci and Pneumococcal Diseases, April 2006, Alice Springs, Australia. Vaccine, 2007, 25:7281-84

Dunbar M, *Moberley S*, Nelson S, Leach A and Andrews R. Clear not simple: An approach to community consultation for a maternal pneumococcal vaccine trial among Indigenous women in the Northern Territory of Australia, Vaccine, 2007, 25(13):2385-8.

Telfer B, *Moberley S*, Hort K, Branley J, Dwyer D, Muscatello D, et al. Probable Psittacosis Outbreak Linked to Wild Birds. Emerging Infectious Diseases. 2005 Mar.

Grants

Associate Investigator on two successful National Health and Medical Research Council of Australia grants (PneuMumII, 2008, grant # 490320 and Immunogenicity of 23-valent pneumococcal polysaccharide vaccination among Indigenous Australian adolescents and adults, grant # 605808).

Training grants

Medicines Australia Vaccines Industry Group Immunisation Award, 2008, Public Health Education and Research Trust, the Public Health Association of Australia.

Public Health PhD Scholarship, March 2006 to Sept 2008, National Health and Medical Research Council of Australia.

PhD Scholarship, March 2005 to March 2006, Centre for Clinical Research Excellence (Indigenous Immunisation).

Trainer

Epidemiology and Biostatistics, Fogarty training program, April 12th to 16th, 2010, Goroka, PNG.
Immunology, About Giving Vaccines, Aboriginal Health Worker Course, June 2005.

Reviewer

British Medical Journal, Vaccine, BMC Infectious Diseases
