# Supplementary material for: Quality improvement intervention to increase adherence to ART prescription policy at HIV treatment clinics in Lusaka, Zambia: A cluster randomized trial
Source: PLoS One. 2017 Apr 18;12(4):e0175534. doi: 10.1371/journal.pone.0175534 (PMC5395211; doi:10.1371/journal.pone.0175534)
Supplement: S2 Protocol — (PDF) [file pone.0175534.s007.pdf]

STUDY PROTOCOL

Version 5.0, 19 Dec 2014

Study Title: Determining the impact of ART pharmacy supply services interventions on the quality and efficiency of ART refills: A pair-matched, cluster-randomised difference-in-difference study

Abbreviated Title: The impact of ART pharmacy supply services interventions on the quality and efficiency of ART refills.

Trial Registration #: To be advised

Principal Investigators:

Dr. Albert Mwango, National ART Coordinator, Ministry of Health

Dr. Sarah Moberley, Senior Technical Advisor, Clinton Health Access Initiative

Co-Investigators:

Mr. Chikuta Mbewe, Deputy Director Pharmaceutical Services, Ministry of Health

Mr. John Ngosa, Director Logistics, Medical Stores Limited

Dr. Bushimbwa Tambatamba, Deputy Director Epidemiology & Disease Control, Ministry of Community Development Mother and Child Health

Dr. Musumba Masaninga, District Medical Officer - Lusaka District, Ministry of Community Development Mother and Child Health

Dr. Leah Namonje, HIV Specialist, Ministry of Community Development Mother and Child Health

Mr. George Kadimba, District Pharmacist – Lusaka District, Ministry of Community Development Mother and Child Health

Helen Bwalya Mulenga, Head Pharmaceutical Services Department,

Center for Infectious Disease Research Zambia
Mpande Mukumbwa-Mwenechanya, POPART Program Manager, Center for Infectious Disease Research Zambia
Jan Willem Van Den Broek, Country Director, Clinton Health Access Initiative
Felton Mpasela, Program Associate (3DE), Clinton Health Access Initiative
Benjamin Chibuye, Program Manager (3DE), Clinton Health Access Initiative
Dr. Marta Prescott, Senior Technical Advisor, Applied Analytics Team, Clinton Health Access Initiative
Hamsa Subramaniam, Research Associate, Applied Analytics Team, Clinton Health Access Initiative
Elizabeth McCarthy, Director, Applied Analytics Team, Clinton Health Access Initiative
Margaret Lippitt, Research Associate, Applied Analytics Team, Clinton Health Access Initiative

Administering Institution: Clinton Health Access Initiative

Dates August 2014 to July 2015

1 PRINCIPAL INVESTIGATOR AGREEMENT

I agree:

- To assume responsibility for the proper conduct of the study;
- To conduct the study in compliance with this protocol, with any future protocol amendments and with any study conduct procedures;
- To ensure that all persons involved with this study are adequately informed about the study-related duties and functions as described in the protocol;
- Not to implement any changes to the protocol without prior review and approval from the IRB approving the protocol;
- That I am aware of, and will comply with "Good Epidemiological Practice" (GEP);
- That I, and any persons employed on this project, will provide up to date curriculum vitaes and any declaration of financial and ownership interests in this project.

Investigator name: DR ALBERT MWANGO

Date: 12-SEPT.2014

2 STUDY SYNOPSIS

Facility congestion is a significant concern in Zambia. With demand for treatment greater than the available capacity of health infrastructure, the MOH and other stakeholders are concerned about the effect that congestion within facilities may have on patient retention in Antiretroviral therapy care, particularly in urban areas.

We intend to conduct a rapid impact evaluation as part of the DFID funded Demand-Driven Evaluations for Decisions (3DE) project. This proposal has been designed in response to the question demanded by the Ministry of Health in Zambia 'How to decongest busy ART clinics in Lusaka.' We seek to generate reliable impact evidence that meets the Ministry's needs and is used to catalyse implementation of cost effective action.

This proposal outlines a study with two components. The first being an intensive assessment period during which time, critical process failures are identified that prevent the provision of 3-month ART refills and the design of an intervention that improves ART service efficiency and improves the supply of ART commodities. The second component is a matched, randomised difference in difference study that will determine the effectiveness of the intervention.

We hypothesise that improvements to ART service efficiency and the quality of pharmacy ART supply will result in an increase in the proportion of patients receiving 3-month refills, which in turn will reduce the frequency of patients visiting the ART clinic and patient wait times.

Table of Contents

1 PRINCIPAL INVESTIGATOR AGREEMENT 3
2 STUDY SYNOPSIS 4
3 GENERAL INFORMATION 7
3.1 Protocol full title 7
3.2 Principal investigators 7
3.3 Person(s) authorised to sign the protocol amendments 7
3.4 Other institutions involved 7
4 AIMS 8
4.1 Objectives 8
5 BACKGROUND AND RATIONALE 9
5.1 Introduction 9
5.2 ART Clinic Congestion 9
5.3 ART refill length 10
5.4 Program description and theory of change 11
5.5 Rationale for Study 11
6 RESEARCH PLAN 12
6.1 Study design 12
6.1.1 Study schema 14
6.1.2 Study population 14
6.1.3 Sampling methodology 14
6.1.4 Primary and Secondary outcomes 15
6.1.5 Inclusion criteria (For both the assessment and evaluation phase) 16
6.1.6 Exclusion criteria 17
6.2 Study procedures 17
6.2.1 Data collection for assessment phase: Service efficiency and quality of pharmacy ART supply service assessment 17
6.2.2 Intervention design 19
6.2.3 Data collection for Evaluation Phase: Assessment of the impact of the intervention 20
6.2.4 Risks and Benefits 21

6.3 Sample size calculation 22
6.4 Primary and secondary analyses 24
6.4.1 Secondary analyses 25
6.4.2 Stopping Rules 25
6.4.3 Dissemination 25
7 ADMINISTRATIVE ASPECTS 26
7.1 Training and Piloting 26
7.2 Supervision and oversight 26
7.3 Monitoring 26
7.4 Recording of data 26
7.5 Data quality control 27
7.6 Confidentiality 27
8 REFERENCES 28
ANNEX 12 Principle Investigator CV Dr Mwango 46
ANNEX 13 Principle Investigator CV Dr Moberley 51

3 GENERAL INFORMATION

3.1 Protocol full title

Determining the impact of ART pharmacy supply services interventions on the quality and efficiency of ART refills: A matched, cluster-randomised difference-in-difference study

3.2 Principal investigators

Dr Albert Mwango, National ART Coordinator, Zambia Ministry of Health

Dr Sarah Moberley, Senior Technical Advisor, Clinton Health Access Initiative

3.3 Person(s) authorised to sign the protocol amendments

Dr. Albert Mwango, Ministry of Health, Dr. Sarah Moberley and Elizabeth McCarthy, Clinton Health Access Initiative

3.4 Other institutions involved

The following study sites in Lusaka District were selected to participate after eligibility assessment:

Bauleni Urban Health Center,Bwafwano Community Urban Health Center, Chazanga Urban Health Center, Chelstone Urban Health Center, George Urban Health Center, Kabwata Urban Health Center, Kalingalinga Urban Health Center, Kamwala Urban Health Center, Kara Clinic, Makeni Urban Health Center, Matero Main Urban Health Center, Matero Referral Urban Health Center, Mtendere Urban Health Center, Ng'ombe Urban Health Center, SOS Medical Center, University of Zambia (UNZA) Health Center.

4 AIMS

The primary aim is to determine the immediate impact of improved service efficiency and quality of pharmacy ART supply on facility-level congestion. To this end facility-level congestion will be measured as the **proportion of stable patients in a three-month period who obtain 3-month refills from the pharmacy**. Stable patients are those who are on first-line treatment or regimen, have been on treatment for more than 6 months, have no health conditions requiring attention of a clinician, have not had any complaints or switched medication in 3-months. Such patients are eligible for a 3 month refill.

The secondary aim is to estimate the effectiveness of improved service efficiency and quality of pharmacy ART supply service on indicators of antiretroviral therapy (ART) outcomes 3 months post intervention:

- Patient wait times
- Average daily clinic attendance
- Average daily load per clinician

Assess the factors that enable the intervention to be successful, including process measures:

- Efficiency of supply chain as measured by stock commodity and health worker perceptions of supply chain reliability (e.g. on time deliveries, adequate storage capacity)
- Patient satisfaction and experiences with transitioning to 3-month refills Health worker perspectives on barriers or challenges to providing 3-month refills
- Compliance to quality improvement standard operating procedures

4.1 Objectives

The primary objectives are:

1. Critically assess the failures and challenges related to service efficiency and quality of pharmacy ART supply service and their role in preventing the provision of 3-month ART refills.
2. Plan and execute a set of interventions based on the assessment of failures and challenges related to facility congestion.

3. After implementation of the interventions, evaluate the impact of the interventions on facility decongestion as measured by the proportion of stable ART patients on 3-month refills in a 3-month time period.

5 BACKGROUND AND RATIONALE

5.1 Introduction

Demand-Driven Evaluations for Decisions (3DE) is a three-year program funded by DFID. The program supports rigorous impact evaluation in areas of utmost importance to the Ministry of Health (MOH) and Ministry of Community Development Mother and Child Health (MCDMCH). It seeks to generate reliable impact evidence that meets the Ministry's needs and is used to catalyse implementation of cost effective action. This protocol is a 3DE Evaluation, and as such, this research question was demanded by the MOH and MCDMCH.

5.2 ART Clinic Congestion

In June 2013, the WHO released guidelines that increase the number of people eligible for antiretroviral therapy (ART) from close to 17 million to more than 26 million people in low- and middle-income countries.[1] This decision reflects significant evidence that increasing treatment coverage and access to high-impact prevention interventions will be a crucial next step in turning the tide of the AIDS epidemic. However, despite significant treatment scale-up over the past decade, countries in Sub Saharan Africa are far from achieving the target of universal access for all those in need and will face numerous challenges in scaling up HIV treatment. In particular, the infrastructure, health system and human resources required to properly provide and monitor treatment are extensive, and current systems have limited capacity to support scaled-up ART programs.[1,2,3] Within Zambia, with the roll-out of strategies to improve access for treatment such as Option B+ and raising the threshold of ART eligibility from 350 CD4 cell count to 500 CD4 cell count, existing ART facilities are crowded and burdened, they will continue to become increasingly congested.

[1] Eligibility under the 2010 Guidelines was originally estimated at 15 M and has since been revised based on changes in the epidemic and modeling. Global Update on HIV Treatment 2013. WHO Report.

Facility congestion is a concern in Zambia where only 450 accredited health facilities provided treatment to approximately 566,000 patients in 2013.[4] Based on the UNAIDS Gap Report, only about one third of HIV positive patients are accessing treatment, suggesting that there are over one million patients[5] in need of ART in Zambia. Although the MOH plans to build 650 health posts that will help to strengthen linkage to care by referring more people to ART facilities as well as having all 1500 PMTCT sites upgraded to ART sites to meet demand, it is essential to identify opportunities for increased efficiencies under current circumstances in order to accommodate these patients. With demand for treatment greater than the capacity within Zambia, the MOH and stakeholders are concerned about the effect that congestion within facilities may have on patient retention in ART care, particularly in urban areas.

Although there is an absence of published literature regarding the implication of congestion on patient retention in ART care, there is evidence to suggest that congestion may affect patient flow and wait times.[6] Moreover, experienced health staff within Zambia have reported that long wait times lead to decreased ART retention, and subsequently, poor treatment outcomes.

5.3 ART refill length

One proxy for facility congestion is the proportion of stable ART patients receiving 3-month refill prescriptions. With longer prescription refill length, as compared to the frequently dispensed 1- or 2-month prescriptions, stable patients are able to space out their facility visits, and thus, reducing the number of people within the facility at any given time. The Zambian ART Guidelines recommend that stable ART patients be provided with prescriptions for up to 3 months at a time,[7] but there is great variation in the degree to which this policy is implemented in health facilities in Zambia.

To assess refill patterns within Zambian facilities, initial assessments were done at five ART clinics in Lusaka. The majority of patients received their ART refill for one month or less (51%), 26% of patients received a 2-month refill and 23% received a 3-month refill. The decision on the ideal refill length is often made by a team (including adherence counsellors and clinicians), however the pharmacist made the final decision based on their knowledge of drug stock levels at the facility. The In-Charge of sampled facilities reported that unreliable stocks of ARTs were the main barrier to the provision of 3-month refills for stable patients.

5.4 Program description and theory of change

With the aim of improving facility-level congestion and based on the results from the preliminary assessment, interventions are being proposed to address facility specific issues centred on clinic efficiency and supply chain quality. A potential theory of change is listed below:

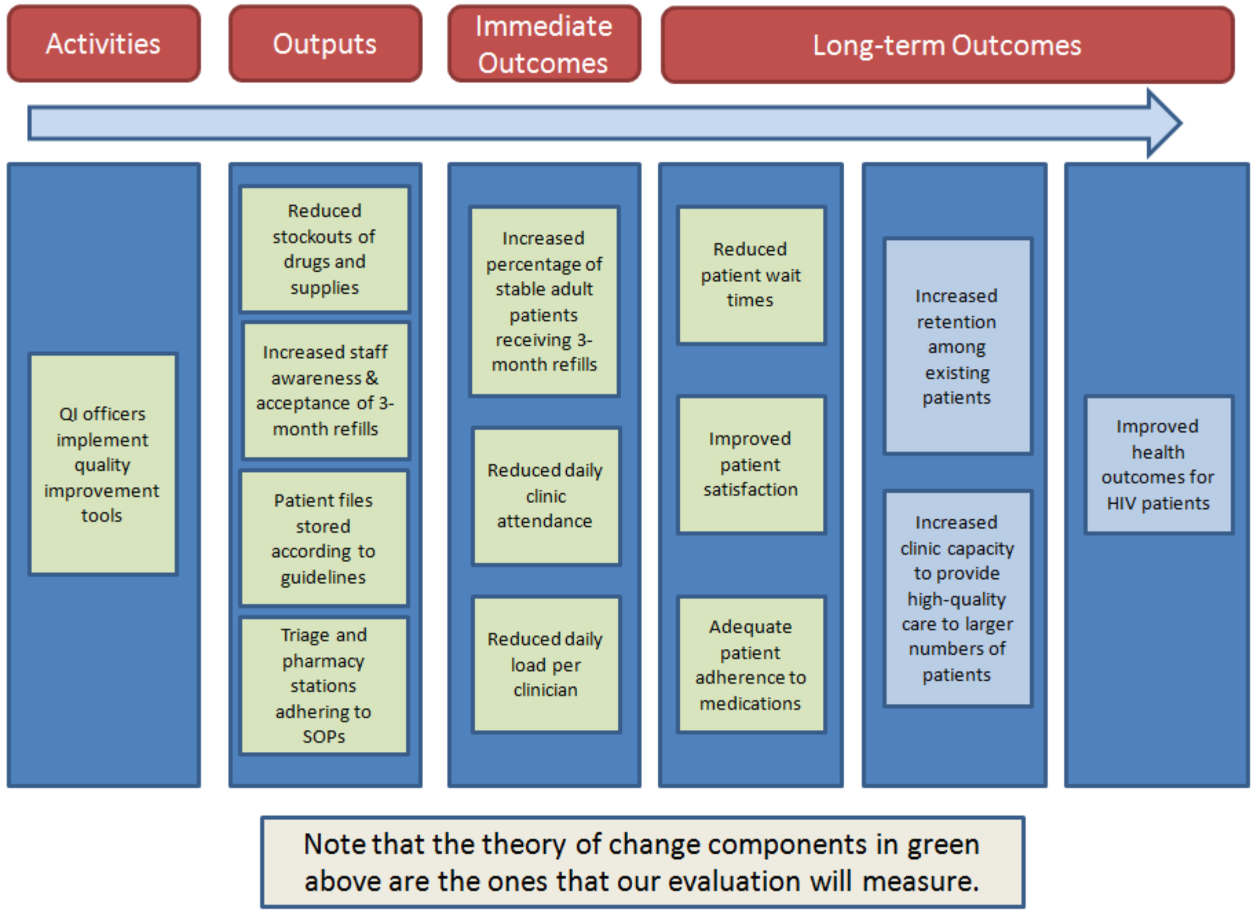

5.5 Rationale for Study

The purpose of this work is to outline the impact of the intervention (developed from the assessment) for ART service efficiency and quality of pharmacy ART supply service on facility congestion as measured by the proportion of stable ART patients on 3-month ART prescriptions. We aim to develop (i.e. Assessment Phase) and test (i.e. Evaluation Phase) service efficiency and pharmacy ART supply chain quality interventions that can be utilized in public, urban facilities throughout Zambia, in order to facilitate the process of scaling up access to ART in Zambia.

6 RESEARCH PLAN

Where applicable the plan is broken down into the components of the assessment phase and evaluation phase.

6.1 Study design

Assessment Phase:

First, we will conduct an assessment of 8 randomly selected facilities and perform an assessment on the current ART service delivery and pharmacy ART supply delivery. The information gathered in this assessment phase will be used to develop the intervention.

Intervention:

1. The assessment data indicated that the primary barrier to stable patients receiving 3-month prescriptions centred around the behaviours of the clinic pharmacist, and more distally, stock availability as well as the perceptions of ART stock availability. Therefore, to identify the added benefit unique to a behavioural intervention, stock would be guaranteed to all participating facilities, thus creating both a basic intervention arm (controls) and the comprehensive intervention. *Basic improvements to all 16 facilities*: As stock availability is a core barrier to administering the 3-month refill prescriptions, all facilities within the program will receive support in ordering and stock planning to guarantee stock levels are sufficient for the patient volumes. Additionally, all facilities within the study will receive a memo to direct staff to follow MOH policy to provide 3-month refills to reduce the frequency of patient facility visits and facility wait times, as well as a package of materials including quality improvement (QI) checklists and facility protocol guides.
2. *Comprehensive improvements to 8 intervention facilities*: Along with reinforcement of stock and awareness of the 3-month prescription policy, the intervention facilities will also have a Facility Quality Improvement Officer who will administer the QI checklists and facility protocol guides. Existing QI protocols will be refined to ensure the research outcomes are addressed. The QI officers will facilitate the completion of checklists, ensure that stock is available at the clinic level for both ART drugs and lab commodities, and troubleshoot problems as they arise. The QI officers will also communicate challenges and progress during frequent and periodic meetings

with CHAI and MOH. In addition, pharmacists will be provided a checklist to promote standardized prescribing behaviours (3-month refills for stable patients).

Evaluation Phase:

After initiating the intervention, we will conduct a cluster randomised, pair-matched, difference-in-difference study to assess the impact of the intervention on the change in proportion of 3-month refill scripts. To assure that the two arms (i.e. the basic intervention and the comprehensive intervention arms) are as comparable as possible, facilities will be matched into pairs based on integration status of facility and the proportion of patients on 3-month refills at baseline. Any additional factors that may make the pairs unbalanced, such as patient loads, will be accounted for in the analysis. After pair matching, facilities will be randomly assigned to the comprehensive intervention; one facility from each pair will be randomly assigned to undergo the intervention with a 2-month period of assessment and baseline data collection, followed by a 3-month period of the intervention, and a final period of data collection obtaining information on the previous 3 months during the intervention. To assess facility-level outcomes, data will be collected in various formats including patient records, patient focus group discussions, and other relevant key informant interviews.

6.1.1 Study schema

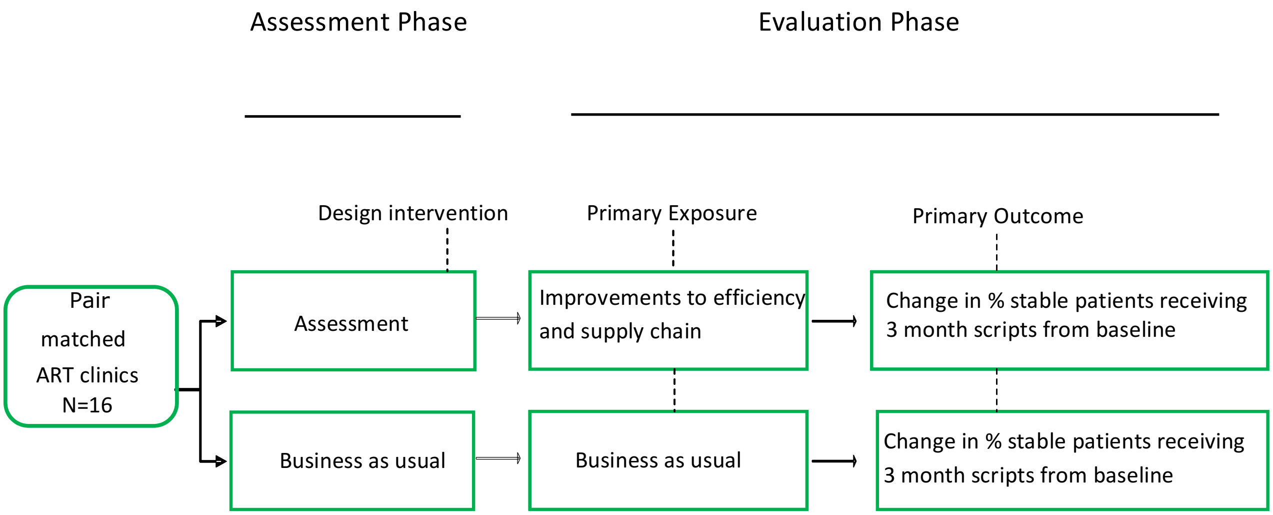

6.1.2 Study population

For this work, the study population will be ART facilities within the Lusaka region of Zambia. Patient study population includes stable adult patients on first-line ART who attend one of 16 selected ART facilities within Lusaka, Zambia from August 2014 to April 2015.

6.1.3 Sampling methodology

There are four types of sampling methods based on the method of data collection: facility, patient & pharmacy records, patient exit interviews, and key informant interviews (including health providers and District and MSL staff responsible for ART supply)

Assessment Phase:

- We will randomly select 16 facilities among all eligible facilities within the Lusaka catchment area and pair-match these facilities. In addition to those randomly selected, some facilities may be included to assure specific key facilities are within the sample for the purposes of policy decisions. From these pairs, we will randomly pick 8 facilities that will receive the intervention, and therefore, participate in the assessment phase of the study.

- One key informant interview and at least one provider interview will be conducted for each facility; these will be selected based on the knowledge base of the facility services and to assure we obtain a range of views and experiences.
- Ten patient exit interviews after the pharmacy will be collected per facility (80 in total) to qualitatively assess the patient view of the facility. Every 10th eligible patient will be referred by the pharmacist. To this end, every 10th patient to leave the facility will be asked to participate in an exit interview. If this patient does not wish to participate or is ineligible, the immediate next participant will be approached.
- A small sample (10 per facility) of ART registries & pharmacy records will be collected per site to better understand the ability to collect data for the evaluation.

Evaluation Phase:

- The 16 facilities (matched in 8 pairs) will be included in the evaluation phase.
- Adult (aged 18 or over) patient records will be collected for all eligible patients who receive care at the facility within the intervention time period as well as the 3-month period prior to baseline.
- For patient wait times, every 10th patient who enters the facility will be followed to monitor their total wait time while in the facility 2 weeks prior to the intervention implementation as well as 2 weeks prior to end line.
- Endline focus group discussions will be held with patients in the intervention facilities to gain a comprehensive understanding of the success of the implementation from the patient perspective.
- Within each facility in the study, at least one clinician (nurse, clinical officer or doctor) and at least one pharmacy staff member will be interviewed to provide facility-level information at both baseline and endline. For staff interviews, participants will be purposefully selected to provide a range of perspectives and experiences.
- Each of the QI Officers will be interviewed at endline.

6.1.4 Primary and Secondary outcomes

Primary Outcome for Evaluation Phase:

The primary outcome is a measure of the change in facility congestion: the change in the proportion of stable ART patients receiving 3-month refills. Specifically, this will be calculated as the change from

baseline to endline in the proportion of 3-month scripts among stable patients within a 3-month window.

Stable patients are those who are on first-line treatment or regimen, have been on treatment for more than 6 months, have no health conditions requiring attention of a clinician, have not had any complaints or switched medication in 3-months, and adhered to visits for the past 6 months. Such patients are eligible for a 3 month refill as outlined in the Zambia Consolidated Guidelines[7] (first-line includes the following combinations (**1**) TDF + FTC/3TC + EFV, (**2**) TDF + FTC/3TC + LPV-r, (**3**) TDF + FTC/3TC + ATV-r, (**4**) TDF + FTC/3TC + NVP+, (**5**) AZT + FTC/3TC + LPV-r, (**6**) ABC + 3TC + LPV-r, (**7**) ABC + 3TC + EFV).

Secondary outcomes for Evaluation Phase include:

- Average patient wait times based on the daily average wait time from clinic entry to exit
- Average daily clinic attendance
- Average daily patient load per clinician

Additional factors assessed to assess success of intervention:

- Patient satisfaction and experiences with transition to multi-month refills
- Stock commodities status (frequency and duration of stock outs)
- Staff perspectives on process of implementing clinical efficiency and supply chain interviews and on barriers or challenges to providing multi-month refills
- Patient, pharmacist and provider perceptions on reliability of pharmacy ART chain

6.1.5 Inclusion criteria (For both the assessment and evaluation phase)

For facility inclusion, facilities are eligible that are within the Lusaka district, are not currently participating in another trial (e.g. popART) and have less than 80% of stable patients on 3 month refills. This last eligibility criteria is to assure that the facilities have the potential to increase the proportion of stable patients on 3-month refills by 20%.

For patient record inclusion, all ART records for stable patients over the age of 18 on first-line treatment who visited the facility in the last 3-months will be examined. To be characterized as stable, a patient must have been on first-line treatment or regimen more than 6 months, has no health conditions

requiring attention of a clinician, has not had any complaints or switched medication in 3-months, and eligible for a 3 month refill as outlined in the Zambia Consolidated Guidelines[7]

For focus group discussions, adult ART patients on first-line treatment and aged 18 years and over will be eligible for the exit interview.

For staff and QI officer interviews, participants must be over the age of 18.

6.1.6 Exclusion criteria

Facilities will be excluded if they are currently participating in research that may interfere with our primary or secondary outcomes.

Patient records will be excluded if they were among those under the age of 18 or were not considered stable.

For focus group discussions, anyone under the age of 18, or those who are not on first line ART will be excluded.

For staff and QI officer interviews, anyone under the age of 18 will be excluded.

6.2 Study procedures

6.2.1 Data collection for assessment phase: Service efficiency and quality of pharmacy ART supply service assessment

The first component of the study will be a detailed assessment of failures in service efficiency and pharmacy ART supply chain. This will include, but not be limited to:

- Facility characteristics:
    - Patient flow assessment will be measured in each site over 2 days recording the patient wait times at each component of the clinic.
    - Daily attendance, facility characteristics, and number of clinicians at the facilities.
    - An assessment of supply chain reports, requisition forms and the flow of supply related reports and commodities.

    - An assessment of any available quality improvement checklist and standard operating procedures related to dispensing ARTs.

- Patient exit interviews to receive information on their experience; what they think works well at the clinic and what does not work well.
- Data collection at the pharmacy dispensing site to identify rationale for refill interval length.
- Data from the ART register that identifies stable patients will be linked to the pharmacy dispensing data in order to determine the baseline proportion of stable patients receiving 3-month refills.
- Key informant interviews of facility, district and MSL staff to determine each member's experiences and cited challenges in the provision of ART care and 3-month refills in particular.

Data collection forms are attached as Annex 1 to 8.

6.2.2 Summary results for assessment phase: Service efficiency and quality of pharmacy ART supply service assessment

In the initial assessment phase of the study, a wide range of data sources were used to assess barriers to 3-month refills and the feasibility of increasing the proportion of stable patients receiving 3-month refills. Patient and provider perceptions of 3-month refills and the barriers to issuing them were explored. Patients verified that clinic congestion is a key barrier to accessing care and that 3-month refills would be preferable from a patient perspective. Providers were mostly in agreement that 3-month refills should be given, but they suggested that staff shortages, poor filing systems, inconsistent drug supplies and misalignment of clinical and lab appointment schedule continue to create challenges for executing this protocol. The data on current refill practices demonstrated a wide variation in practices across facilities, but the majority of patients were receiving 1 or 2-month refills. Data on drug supplies verified that stockouts could possibly be one of the barriers leading to limited 3-month refills. From Jan-Oct 2014, 3 of 8 facilities experienced 1-3 stockouts of TDF-FTC-EFV (Atripla) the fixed-dose combination (FDC) regimen recommended for stable first-line patients. The average duration of Atripla stock-out ranged from 1 to 4.5 days, average of 2.5 days. Finally, patient wait times indicated that the average time spent at the clinic for any visit was just short of 2 hours, while pharmacy-only visits were on average an hour and a half. These times exclude the wait time prior to triage, spent in line often starting early in the morning before the clinic opens.

6.2.3 Intervention design

The intervention arm of the RCT will undergo a two-part phase: an assessment phase and then the intervention designed based on the assessment. The design of the intervention will be informed by the above assessment. In particular, critical process failures will be identified and an intervention designed to resolve the issue. A stakeholder meeting will take place and involve all relevant parties including Facility in-charges, District Pharmacists and ART Coordinators, MSL staff responsible for ART supply, and collaborators. The purpose of this stakeholder meeting is to ensure that there is buy-in and ownership of the intervention from all relevant stakeholders.

6.2.4 Data collection for Evaluation Phase: Impact of the intervention

Based on the randomized pair-matched study design, ongoing assessments will measure process and outcome indicators (outlined below).

TOC Stage	Component	Baseline	Endline	Measurement Source
**Activities**	QI officer implementation of quality improvement tools		✓	Collection of QI officer checklists, facility staff interviews (intervention sites only)
**Outputs**	Frequency and duration of stock-outs of drugs and supplies	✓	✓	Facility stock data
	Level of staff awareness of 3-month refills		✓	Facility staff interviews (intervention sites only)
	Level of compliance of facility with patient files storage guidelines		✓	Collection of QI officer checklists, facility staff interviews (intervention sites only)
	Level of compliance of triage and pharmacy stations with SOPs		✓	Collection of QI officer checklists, facility staff interviews (intervention sites only)
**Immediate Outcomes**	Percentage of stable adult patients receiving 3-month refills	✓	✓	CIDRZ SmartCare data
	Average daily clinic attendance	✓	✓	Facility registry data
	Average daily load per clinician	✓	✓	Facility registry data
**Long-term Outcomes**	Patient wait times	✓	✓	Observations of patient visits
	Patient satisfaction		✓	Focus group discussions
	Level of self-reported adherence		✓	Focus group discussions

As facilities and caregivers cannot be blinded to the intervention they are receiving due to the behavioral nature of the likely intervention, we will only be able to mask the type of intervention on the patients themselves. Additionally, the data collectors as well as the analysts will be blinded. For

quality purposes, however, certain study staff will know the intervention facilities in order to describe and implement the intervention.

6.2.5 Risks and Benefits

Risks: The risks of this study relate only to access of identifiable information. As outlined above the study team will go to great lengths to ensure patient confidentiality is maintained. All staff will have been trained and committed to maintain confidentiality. All records will be de-identified and will be stored in a password protected database and any original Case Report Forms will be stored in a locked filing cabinet within a locked room at the Clinton Health Access Initiative Office in Lusaka.

We will ask permission to record the interviews for quality control purposes. These recordings will also be confidential and be stored in a password-protected database once transferred, and immediately deleted from the recording device. In cases where the client declines to being recorded, the data collector will seek consent to interview the client while only taking notes. All recording devices will be password protected such that only team members can open the device. Overall, the study does not pose any clinical related risks to individual study participants. Only stable patients who have been on treatment for more than 6 months, have had no health conditions requiring attention of a clinician and have not had any complaints or switched medication in 3-months will be eligible to participate.

Benefits: The benefits of the study will be for improved efficiency measures being rolled out across all sites should the Government decide that the intervention was successful. At individual level, some of the indirect benefits to study participants will include; better patient experiences by receiving 3 month refills and reduced number of visits to the ART clinics once put on longer refills.

No compensation will be provided to participants of surveys other than refreshments.

Autonomy: To assure all participants are aware of these risks and benefits, informed consent will be sought from each key informant or patient exit interview participant in the assessment and evaluation phase in accordance with IRB approval following an explanation of the study using a Plain Language Statement approved by the IRB. Study staff will clearly explain the rights of potential participants to decline to participate at any point without any negative consequence. Additionally, the consent form will obtain permission to record the conversation. Each participant will also be given a copy of the study

information sheet which clearly explains the risk and benefits of the study. The information sheet will also have contact details of the contact person from the study team and ERES IRB.

All consent forms and patient information sheets are attached to this application as Annex 8 -10. A minimal dataset summarising the outcomes from those approached for the Plain Language Statement will be recorded through a Screening Log.

Participant questionnaires approved by the IRB in electronic or paper format will be used to document patient wait times, information relating to the barriers/influences of being retained in ART care, patient and facility staff identified challenges regarding the provision of longer refills, and select socio-demographic indicators.

To assure that ethical guidelines are followed, this study will be conducted according to Good Epidemiological Practises.[8] Staff will be required to have undergone training in ethics and the management of study records according to Standard Operating Procedures. Additionally, CHAI study team members will be present in the field during data collection to oversee and monitor that the protocol and guidelines are followed.

In the event that any of the ethical considerations are not followed, or in the case of an adverse event (e.g. informed consent not obtained), the study team will report to the study team leads who will then report to IRB as appropriate

6.3 Sample size calculation

Assessment Phase: As the assessment will be a qualitative assessment of the service delivery and pharmacy ART supply quality, our sample size is based on obtaining enough qualitative information to create proper interventions. For the assessment 8 facilities will be selected; within the each of the facilities 10 ART & pharmacy registries per facility, 10 patient exit interviews, 1 key informant, and 1 provider will be selected.

Evaluation Phase: For the pair-matched, cluster-randomized design, we will collect all available ART registries from the facilities for eligible patients & pharmacy records at baseline and endline. The sample size of 16 facilities (estimated 2300 patients per facility with a 20% potential for missing records – an estimate of 1840 per facility)[9] over the 3-month recruitment period will have a power of 80% (α=0.05) to detect difference of ≥17% for the change in the number of patients receiving 3-month refills in the

proceeding 3-month period in the intervention group against the change in the control group, assuming a change of 5% in the control group due to the simple intervention alone (Hayes & Bennett, 1999). The sample size calculation was performed according to the following equation for the comparison of two proportions:

$$c = 2 + \left(z_{\frac{\alpha}{2}} + z_{\beta}\right)^{2} \frac{[\pi_0(1-\pi_0) + \pi_1(1-\pi_1) + (1+(m-1)\rho)]}{m(\pi_0 - \pi_1)^2}$$

The equation was calculated according to the following parameters:

Parameter	Value
Alpha (α)	0.05
Power (β)	0.84
Number of patients per cluster (m)	1840
Estimated change in proportion of 3-month refills (π_0)	17%
Estimated change in proportion of 3-month refills in control (π_0)	5%
Rho	0.1
c (per arm, treatment and control groups)	8
J (total number of groups)	16

For clinic flow observations, we determined that with 20 patients selected at baseline and endline, we would have 80% power to detect a 20 minute difference in the means of the pharmacy wait times between baseline and endline for the intervention and control groups. This was estimated assuming that conservatively, the mean wait time for patients was 47 minutes.

Parameter	Value
Alpha (α)	0.05
Number of patients per cluster (m)	20
Mean wait time in control	47 minutes
Estimated mean wait time in intervention	2.5
Rho	0.4

J (total, treatment and control groups)	16
Power (β)	0.80

6.4 Primary and secondary analyses

First, to assess the ability of the matching to produce comparable groups, the characteristics of the matching factors will be compared between the control and intervention group at baseline. Second, to assess the presence of other potential confounders, facility-level factors such as the age distribution of the patients or proportion of the population female will be compared between the two groups. Bivariable comparisons will also compare how these potential confounders are associated with the primary outcome. After examining the success of the matching and the existence of other potential confounders, the remaining analyses will continue with matched analytical methods. If the groups are unbalanced after matching, we will break the pairs to account for the baseline proportion of 3-month patients in multivariable analysis as this is considered the more efficient method for analysis (cite Hayes).

The primary analysis will be a pair-matched comparison of cluster-level summaries. It will examine the difference between the intervention and non-intervention facilities in the change (from baseline – endpoint) of the proportion of eligible patients in the previous 3-month period who obtained 3-month refills from the pharmacy, adjusted for relevant indicators. The denominator for baseline and endline will include all stable patients active in care per facility over the previous 3-months from the date of data collection. We will compute all the paired comparisons as well as the overall estimate of the difference in the change in 3-month prescriptions, using weighted values to account for varying cluster (i.e. facility) sizes. For tests of significance, we will use pair matched methods (weighted t-test) to compare the difference between the proportions between the matched pairs and will compare these results to non-parametric tests for more robust estimates due to the small number of pairs (8). For further adjustments for potential confounders, conditional multivariable models will be run to examine the difference in the proportion of increased 3-month refills between the intervention and the non-intervention groups.

If matched pairs need to be broken due to lack of appropriate balance at baseline, we will also carry out individual-level analysis of the clustered data to see if the probability of receiving 3-month refills is greater in intervention facilities than control facilities 3-months after the intervention. To this end, we

will run generalized estimating equations (GEE) to estimate the difference as well as account for facility-level characteristics including baseline refill practices.

6.4.1 Secondary analyses

Secondary analyses will consider the impact of the intervention on patient wait times between the intervention and control sites. We will run similar cluster-level summaries and compute weighted overall differences. As with the primary analysis, parametric tests (paired t-test will be used to examine the differences between the two groups for the variable of patient wait times and other secondary outcomes; non-parametric methods will also be considered. Conditional multivariable analyses will be used to account for matched analyses as well as potential confounders. Audio recordings from the qualitative methods, including patient focus groups, staff interviews and QI Officer interviews, will be transcribed. One or more staff members will read each transcript and code the information based on the topics discussed by participants. The code structure will be developed based on directly on the topics emerging in interviews and focus groups. The final results of this analysis will be presented as a set of key themes and ideas, along with representative quotations. Finally, we will describe compliance to the intervention, based on the collection of QI checklists from QI Officer.

6.4.2 Stopping Rules

Midline analyses will be conducted and the study will cease should there be evidence of a beneficial effect of the intervention of a least a 20 percent difference between the intervention and control sites.

6.4.3 Dissemination

The work from this impact evaluation will be shared directly with the Government of the Republic of Zambia and stakeholders to inform the potential scale up of the proposed intervention. Additionally, the results from this work will be shared with community audiences who participated in the work by presenting findings to the facilities and all key stakeholders where appropriate.

7 ADMINISTRATIVE ASPECTS

7.1 Training and Piloting

All interviewers will be trained in data collection methods and ethical guidelines. Additionally, the study tools and data collection documents will be piloted in a facility not selected for the study.

7.2 Supervision and oversight

Due to the sensitive nature of the ART population, the Principle Investigators will closely supervise on and provide oversight to multiple aspects of the study.

- The PIs will work closely with the study co-ordinator to assure that all aspects of the protocol are followed. If there is evidence of any deviation from the protocol, the PIs will be responsible for reporting deviations to the IRB board and taking appropriate actions.
- In the case of adverse events due to the study, the PIs will work closely with the study co-ordinator to address any concerns and raise applicable occurrences to the IRB board.
- The PIs will closely supervise the implementation aspects of the study by monitoring the recruitment of the study population, the attainment of the appropriate sample size, and approving proper adjustments if necessary.
- The PIs will be responsible for the analysis, interpretation, and dissemination of the findings as well as creating a plan for scale up of the intervention, if successful, within Zambia

7.3 Monitoring

Study monitoring will be the responsibility of the Study Coordinator, Felton Mpasela of the Clinton Health Access Initiative, and will be conducted according to standard operating procedures.

7.4 Recording of data

Source data will be collected in study workbooks, either in paper format or in electronic form with direct data entry utilising tablets. All data will be entered into a secure electronic database. The Case Report

Form (CRF) will be an electronic data file compromised of core data fields. Data will be entered directly into electronic CRFs via a secure web interface. All entries on the CRF must be backed up by source data, unless there is a note to file specifying a deviation from this requirement for a specific purpose.

Workbooks and source data must be kept in order and up-to-date so that they always reflect the latest observations on the participants enrolled in the study. Entries into workbooks must be legible and any changes or corrections to the workbook should not obscure the original entry. Handwritten entries are to be done using a non-soluble/non-smudging ball point pen. Errors are to be corrected by passing a single line through the error and accompanied by the researcher's initials and date. White-out fluid is not to be used under any circumstance. Signature logs of all study staff are to be retained in the Central Investigator files.

7.5 Data quality control

Following completion of each component of the CRF, the data will be checked for consistency, logic and range, either through the process of direct data entry into an eCRF or through analyses of the study database. Queries will be generated for spurious data and clarification sought in writing from the Principle Investigator or delegate. Data query forms will then be forwarded back to the staff responsible for database amendment. If necessary, source documents may need to be accessed to correct data errors.

7.6 Confidentiality

All identifiable information on study patients will be retained in password protected files and locked cabinets at study sites. Access to this information will only be provided to immediate study staff who would have signed confidentiality agreements. No identifying information will be included in study reports.

8 REFERENCES

1. Kober K, Van Damme W. Scaling up access to antiretroviral treatment in southern Africa: who will do the job? Lancet. 2004;364(9428):103–107.
2. Van Damme W, Kober K, Laga M. The real challenges for scaling up ART in sub-Saharan Africa. AIDS.2006;20:653–656
3. Jaffe HW. Universal access to HIV/AIDS treatment: promise and problems. JAMA. 2008;300:573–575.
4. ART report, Zambian Ministry of Health, Quarter 3 of 2013.
5. UNAIDS. The Gap Report. 2014. http://www.unaids.org/en/media/unaids/contentassets/documents/unaidspublication/2014/UNAIDS_Gap_report_en.pdf
6. Wanyenze R, Wanger G, Alamo S, Amanyire G, Ouma J, Kwarisima D, Sunday P, Wabwire-Mangen F, Kamya M. Evaluation of the Efficiency of Patient Flow at Three HIV Clinics in Uganda. AIDS Patient Care STDS. Jul 2010; 24(7): 441–446.
7. Zambian Ministry of Health and the Ministry of Community Development Mother and Child Health. Zambia Consolidated Guidelines for the Treatment and Prevention of HIV Infection. February 2014.
8. International Epidemiological Association, Guidelines for Proper Conduct in Epidemiological Research, November 2007, http://ieaweb.org/good-epidemiological-practice-gep/, accessed 30th July, 2014.
9. Scott CA, Iyer HS, McCoy K, Moyo C, Long L, Larson BA1, Rosen S. Retention in care, resource utilization, and costs for adults receiving antiretroviral therapy in Zambia: a retrospective cohort study. BMC Public Health. 2014 Mar 31;14:296. doi: 10.1186/1471-2458-14-296.
